# Biocombinatorial Synthesis of Novel Lipopeptides by COM Domain-Mediated Reprogramming of the Plipastatin NRPS Complex

**DOI:** 10.3389/fmicb.2016.01801

**Published:** 2016-11-17

**Authors:** Hongxia Liu, Ling Gao, Jinzhi Han, Zhi Ma, Zhaoxin Lu, Chen Dai, Chong Zhang, Xiaomei Bie

**Affiliations:** ^1^College of Food Science and Technology, Nanjing Agricultural UniversityNanjing, China; ^2^College of Life Sciences, Nanjing Agricultural UniversityNanjing, China

**Keywords:** plipastatin, biosynthetic complex, COM domain, lipopeptide, NRPS

## Abstract

Both donors and acceptors of communication-mediating (COM) domains are essential for coordinating intermolecular communication within nonribosomal peptides synthetases (NRPSs) complexes. Different sets of COM domains provide selectivity, allowing NRPSs to utilize different natural biosynthetic templates. In this study, novel lipopeptides were synthesized by reprogramming the plipastatin biosynthetic machinery. A Thr-to-Asp point mutation was sufficient to shift the selectivity of the donor COM domain of ppsB toward that of ppsD. Deletion and/or interchangeability established donor and acceptor function. Variations in acceptor COM domain did not result in novel product formation in the presence of its partner donor, whereas plipastatin formation was completely abrogated by altering donor modules. Five novel lipopeptides (cyclic pentapeptide, linear hexapeptide, nonapeptide, heptapeptide, and cyclic octapeptide) were identified and verified by high-resolution LC-ESI-MS/MS. In addition, we demonstrated the potential to generate novel strains with the antimicrobial activity by selecting compatible COM domains, and the novel lipopeptides exhibited antimicrobial activity against five of the fungal species at a contention of 31.25–125 μg/ml.

## Introduction

**Figure 1:**
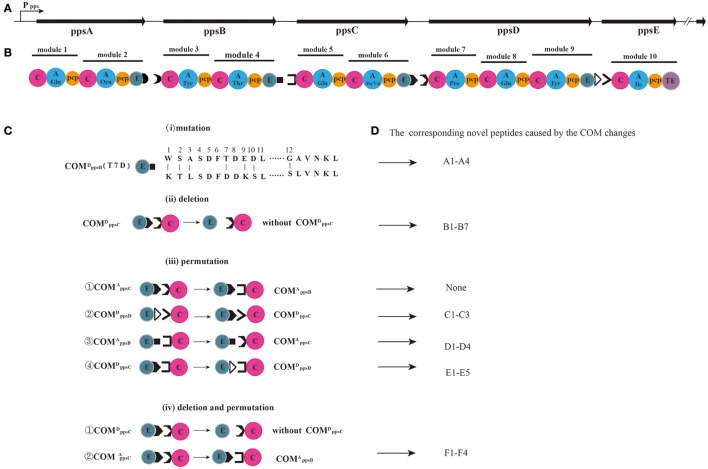
**The plipastatin biosynthetic system. (A)** The enzymatic assembly line of plipastatin consists of five NRPSs, encoded by the polycistronic genes *ppsABCDE*. **(B)** The synthetases *ppsA, ppsB, ppsC, ppsD*, and *ppsE* are composed of two, two, two, three, and one module(s), respectively. **(C)** Variations of COM domains: (*i*) point mutation of donor ppsB; (*ii*) deletion of donor ppsC; (*iii*) four permutations: the acceptor of ppsC replaced with ppsB; the donor of ppsD replaced with ppsC; the acceptor of ppsB replaced with ppsC; and the donor of ppsC replaced with ppsD; (*iv*) the deletion of donor ppsC and the acceptor of ppsC replaced with ppsB. **(D)** The novel peptides caused by the COM changes corresponding to the lipopeptides in Table 1.

*Bacillus subtilis* is a Gram-positive bacterium that produces broad spectrum amphiphilic lipopeptides with excellent biosurfactant properties and antifungal, antibacterial and antiviral activities (Marahiel et al., 1997; Schwarzer et al., 2003; Batool et al., 2011). Iturins (Hiradate et al., 2002; Yu et al., 2002; Jin et al., 2014), fengycins (Vanittanakom et al., 1986; Guo et al., 2014) and surfactins (Bonmatin et al., 2003; Liu et al., 2015) are the three most well-known families of lipopeptides, which produced by *Bacilli*. And all of them are synthesized by nonribosomal peptide synthetases via a thioesterase chain release mechanism (Tosato et al., 1997; Finking and Marahiel, 2004; Calcott and Ackerley, 2014). These lipopeptides contain a variable cyclic amino acid portion attached to a variable β-amino or β-hydroxy fatty acid (de Faria et al., 2011). The fengycin is actually identical compounds to plipastatin that display slightly structural variations at different salty conditions (Honma et al., 2012). Therefore, the term plipastatin is used throughout this study. Plipastatin is composed of 10 α-amino acids linked to one unique β-hydroxy fatty acid chain, and two variants (plipastatin A and B) with Val or Ala at position 6 have been reported (Vater et al., 2002; Sun et al., 2006). Plipastatin synthetase contains five distinct NRPSs subunits which assemble to form a co-linear chain in the order of ppsA-ppsB-ppsC-ppsD-ppsE (Figure 1A) that incorporates two (ppsA, B and C), three (ppsD), and one (ppsE) amino acid residues (Figure 1B) into the growing peptide, respectively (Marahiel et al., 1997).

Based on the molecular mechanisms employed by NRPSs, NRPS assembly lines have an enormous potential for biocombinatorial synthesis. Biosynthesis of a defined, full-length product relies on the selectivity of individual modules and their coordinated interplay with donor and acceptor communication-mediating (COM) domains (Chiocchini et al., 2006). In most bacterial NRPS systems, a donor COM domain (${\text{COM}}_{\text{X}}^{\text{D}}$) situated at the C terminus of an aminoacyl- or peptidyl-donating NRPS (X) and an acceptor COM domain (COM^A^) located at the N terminus of the accepting partner enzyme (Y) form a matching (compatible) set that forms productive interactions between adjacent modules (i.e., ppsA ppsB1 and ppsB2 ppsC1) (Hahn and Stachelhaus, 2006). Biochemical investigations on the selectivity of NRPSs have helped define sets of compatible inter-module linkers. In principle and in practice, enzymes of a NRPS complex can form other biosynthetic templates, making possible the synthesis of a vast array of novel peptide products via a process that is tantamount to combinatorial synthesis (Sieber and Marahiel, 2005; Fischbach and Walsh, 2006). The COM domain swapping experiments verified the decisive role of COM domains for the control of protein-protein interactions between surfactin NRPS (Chiocchini et al., 2006). The research had demonstrated that point mutation of one of these key residues within the COM domain of TycC1 was sufficient to shift its selectivity from the cognate donor COM of TycB3 toward the noncognate donor COM domain of TycB1 in tyrocidine NRPSs (Hahn and Stachelhaus, 2006). The fragments of COM domains were sequenced in previous study (Hahn and Stachelhaus, 2006), but the interaction between enzymes of a nonribosomal peptide has hardly been studied, which relies on the interplay of COM domains. Therefore, we attempted to investigate the biocombinatorial synthesis of novel lipopeptides by COM domain-mediated reprogramming of the plipastatin NRPS complex.

The goal of this study was to investigate the influence on protein–protein communication based on the converting donor and acceptor of COM domains sequences, which maintain or prevent the selective interaction between partner NRPSs, furthermore the influence on the novel lipopeptides formation. In this work we began with the *B. subtilis* PB2-L strain from our previous work, which produces plipastatin (Figure S5) following integration of a gene expression frame composed of a constitutive promoter P43, functional gene *sfp*, and regulatory gene *degQ* into the chromosomal *amyE* locus of PB2. We studied the importance of COM domains in mediating the specific channeling of reaction intermediates between partner enzymes. First, the effect of site directed mutagenesis of module ppsB on protein-protein communication with the partner elongation module ppsC was explored. Subsequently, the importance and generality of COM domains was further substantiated by COM domain deletion and permutation experiments. This research may provide the theoretical basement for the biocombinatorial synthesis of NRPS complex and the exploitation of novel lipopeptides.

## Materials and methods

### Strains, media, and general methods

Bacterial strains and plasmids used in this work are listed in Table S1. *Escherichia coli* and *Bacillus subtilis* were grown in LB medium supplemented with 100 mg/ml ampicillin, 20 mg/ml kanamycin, or 5 mg/ml erythromycin (final concentrations) where applicable. All enzymes were commercial preparations and were used as specified by the suppliers (Vazyme Biotech Co., Ltd, Nanjing, China and Takara Shuzo Co., Ltd, Dalian, China). *E. coli* transformation was performed as described previously (Sambrook et al., 1989). The plipastatin biosynthetic complex was reprogrammed using a homologous recombination approach (Figure S1) with upstream (A1) and downstream (A2) primers to construct plasmid pks2A1A2. Similarly, upstream B1 (targeting the *tgf* gene fragments) and downstream B2 were used to generate plasmid pks2B1B2. The constructs was verified by PCR analysis and used for deletion and substitutions, respectively. After transformation of the plasmids into the host, plasmids were inserted into the chromosome by homologous recombination between the target gene and homologous sequences, or between the homologous sequences alone via the well-established two-step exchange method (Chiocchini et al., 2006). Integration into the chromosome by a single crossover event was selected during growth at the nonpermissive temperature (37°C) while maintaining selective pressure. Subsequent growth of the cointegrates at the permissive temperature (30°C) leads to a second recombination event, resulting in their resolution (Arnaud et al., 2004). The novel *B.subtilis* strains were verified by PCR analysis and the sequences were sequenced by Genscript biotechnology co., LTD (Nanjing, China) (data not shown).

### Cloning

*Bacillus subtilis* DNA was isolated from enrichment cultures using a bacterial genomic DNA extraction kit (Omega, USA) according to the manufacturer's instructions. Each 50 μl reaction contained 35 μl of ddH_2_O, 5 μl of 2 × PCR buffer, 4 μl of dNTP, 2 μl of F-primer, 2 μl of R-primer, 1 μl of Phanta Super-Fidelity DNA Polymerase and 1 μl of template DNA. The PCR program consisted of an initial denaturation at 94°C for 3 min, followed by 30 amplification cycles of 94°C for 30 s, 60°C for 30 s, and 72°C for 30 s, then 72°C for 10 min. Primers (Supplemental Data) were purchased from Sangon Biotech (Shanghai, China). Standard procedures were applied for all DNA manipulations (Sambrook et al., 1989).

A 1382 bp product containing the COM_ppsB_ fragment was amplified using oligonucleotides ppsB-F and ppsB-R. The resulting product was digested with *Sph*I and *Sal*I and cloned into the *E. coli* expression vector PMD-19 that was digested with the same enzymes to give pCC42. After digestion with *Sph*I and *Sal*I, the fragment was ligated into pKS2 to give pKSA1. Based on the 21 bp overlapping region in M-F and M-R, pKSA1 was used as template for the subsequent PCR with oligonucleotides M-F and M-R to generate point mutations.

Two 600 bp ppsC fragments were amplified using oligonucleotides K-up-F and K-up-R, and K-down-F and K-down-R, respectively. After cloning into the *E. coli* expression vector PMD-19 and digestion with *Cla*I, *Sal*I, *Sal*I, and *Kpn*I, the fragments were ligated into pKS2 to give pKSK1, which was used as template for PCR with oligonucleotides P1-F and P1-R to gain the pKSP1 for use in the initial substitution step. Subsequently, 650 bp upstream and downstream gene fragments were amplified by PCR and the target ${\text{COM}}_{\text{ppsC}}^{\text{D}}$ (the donor COM of ppsC) fragment was combined with the downstream fragment by fusion PCR using the oligonucleotides P2-down1-F and P2-down2-F. The resultant product was digested with *SalI* and *Kpn*I and ligated into pKS2 that was also digested with these enzymes, and oligonucleotides P2-up-F and P2-down-R were used to generate pKSP2. Next, PCR was performed using oligonucleotides P3-F and P3-R and pKSK1 as template to generate pKSP3 for permutation in the third step of the procedure. To this end, 650 bp upstream and downstream fragments were amplified using oligonucleotides P4-up-F, P4-up-R, P4-down3-F, and P4-down-R, while a 72 bp fragment of ${\text{COM}}_{\text{ppsD}}^{\text{D}}$ was amplified using oligonucleotides P4-down1-F and P4-down2-F. After purification, DNA fragments were combined and used as template for PCR amplification with oligonucleotides P4-up-F and P4-down-R, and subsequent cloning into pKS2 yielded the final disruption vector pKSP4 (Table S1). The maps of plasmids used to construct the deletion mutant, point mutation and substitution were shown in Figures S3, S4, respectively.

### *B. subtilis* strain construction

Transformations were carried out as described previously (Anagnostopoulos and Spizizen, 1961), and genotypes were verified by PCR. The pKSA1, pKSK1, and pKSP1 plasmids were transformed into *B. subtilis* PB2-L, *B. subtilis* PB2-LP1 was transformed with pKSP2, and the resulting strain was transformed with pKSP3 and pKSP4, generating *B. subtilis* mutants PB2-LA1 (${\text{COM}}_{\text{ppsB}}^{\text{D}}$T::D), PB2-LK1 (Δ${\text{COM}}_{\text{ppsC}}^{\text{D}}$), PB2-LP1 (${\text{COM}}_{\text{ppsC}}^{\text{A}}$:: ${\text{COM}}_{\text{ppsB}}^{\text{A}}$), PB2-LP2 (${\text{COM}}_{\text{ppsC}}^{\text{A}}$:: ${\text{COM}}_{\text{ppsB}}^{\text{A}}$ and ${\text{COM}}_{\text{ppsD}}^{\text{D}}$:: ${\text{COM}}_{\text{ppsC}}^{\text{D}}$), PB2-LP3 (${\text{COM}}_{\text{ppsC}}^{\text{A}}$:: ${\text{COM}}_{\text{ppsB}}^{\text{A}}$, ${\text{COM}}_{\text{ppsD}}^{\text{D}}$:: ${\text{COM}}_{\text{ppsC}}^{\text{D}}$ and ${\text{COM}}_{\text{ppsB}}^{\text{A}}$:: ${\text{COM}}_{\text{ppsC}}^{\text{A}}$), and PB2-LP4 (${\text{COM}}_{\text{ppsC}}^{\text{A}}$:: ${\text{COM}}_{\text{ppsB}}^{\text{A}}$, ${\text{COM}}_{\text{ppsD}}^{\text{D}}$:: ${\text{COM}}_{\text{ppsC}}^{\text{D}}$, ${\text{COM}}_{\text{ppsB}}^{\text{A}}$:: ${\text{COM}}_{\text{ppsC}}^{\text{A}}$ and ${\text{COM}}_{\text{ppsC}}^{\text{D}}$:: ${\text{COM}}_{\text{ppsD}}^{\text{D}}$). The pKSK1 and pKSP1 plasmids were transformed into *B. subtilis* PB2-L in two steps to give *B. subtilis* mutants PB2-LK1 (Δ${\text{COM}}_{\text{ppsC}}^{\text{D}}$) and PB2-LKP1 (Δ${\text{COM}}_{\text{ppsC}}^{\text{D}}$, ${\text{COM}}_{\text{ppsC}}^{\text{A}}$:: ${\text{COM}}_{\text{ppsB}}^{\text{A}}$) (Table S1).

### Production of antimicrobial extracts

Modified strains were cultivated in Landy medium (20 g/L glucose, 1 g/L yeast extract, 5 g/L L-glutamic acid, 1 g/L KH_2_PO_4_, 0.16 mg/L CuSO_4_, 0.5 g/L MgSO_4_·7H_2_O, 0.15 mg/L FeSO_4_, 0.5 g/L KCl, 5 mg/L MnSO_4_·H_2_O, pH 7.0) at 33°C in a rotary shaker at 180 rpm for 3 days to produce antimicrobial substances (Landy et al., 1947; Deng et al., 2011b). Antimicrobial extracts were obtained using an organic solvent (methanol) extraction method.

### Product formation assays

The crude methanol extract was fractioned by the high resolution LC-ESI-MS and LC-ESI-MS/MS analysis, which was performed with a Thermo Finnigan Surveyor-LCQ DECA XP Plus (Thermo Electron Corporation, San Jose, CA, USA). The flow rate was maintained at 0.2 mL/min with a gradient of 22 min (0–95 %, vol/vol acetonitrile for 18 min and 95–5%, vol/vol acetonitrile for 4 min). The electrospray needle was operated at a spray voltage of 5 kV, a capillary voltage of 32 V and a capillary temperature of 300°C. For the HCD experiment, helium was used as the collision gas and the collision energy was set at 35%. Elution was monitored by UV detection at 214 nm (Mootz et al., 2000; Deng et al., 2011b).

### Determination of antimicrobial activity

The linear products were synthetized by KareBay BioChem, Inc. Antimicrobial activity was evaluated by determining MIC values using the standard broth microdilution method (Nedorostova et al., 2009) with some modifications. Tested microorganisms were incubated at 37 or 30°C to approximately 10^6^–10^7^ CFU/ml in lysogeny broth (LB) medium. Serial dilutions of antimicrobial compounds were prepared to obtain final concentrations of 1000, 500, 250, 125, 62.5, 31.25, 15.63, and 7.81 μg/ml in LB or PDA medium. The ability of cyclic products strains to inhibit the growth of various indicator organisms was determined qualitatively by agar-well diffusion method (Deng et al., 2011a). Plates were incubated for up to 24 (bacteria) and 48 h (fungi) before the antimicrobial activities were determined (Oh et al., 2011). Samples incubated without antimicrobials were used as controls.

## Results

### Influence of a ppsB donor point mutation on plipastatin synthesis

To demonstrate the biocombinatorial potential of COM domains, we investigated crosstalk between ppsB and ppsC modules through point mutations. Seven positions in ${\text{COM}}_{\text{ppsB}}^{\text{D}}$ (Figure 1C) were mutated as follows: W1K, S2T, A3L, T7D, E9K, D10S, and G12S. We evaluated the impact of ${\text{COM}}_{\text{ppsB}}^{\text{D}}$ point mutants on lipopeptide biosynthesis.

**Figure 2:**
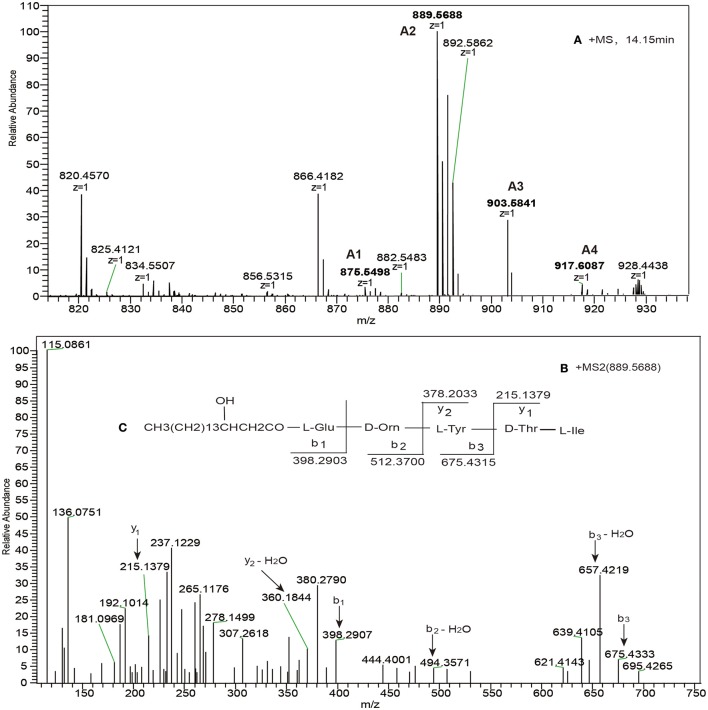
**(A)** The high-resolution ESI–MS of cyclic pentapeptide ions with retention time (RT) 14.15 min and **(B)** HCD–MS/MS of the precursor ion [M + H]^+^ at *m/z* 889.5688, **(C)** which was confirmed as a novel pentapeptide consisting of Glu-Orn-Tyr-Thr-Ile and a C_13_ β-OH fatty acid chain.

Based on the high-resolution LC-ESI–MS spectra, all the point mutants did not produce novel lipopeptides (data not shown) except for the T7D mutation. In high-resolution ESI–MS spectra, a series of charge 1 (*z* = 1) molecular ions with *m/z* values at 875.5488, 889.5644, 903.5801, and 917.5963 (Figure 2A) led us to predict the formation of a β-OH fatty acid (β-OHFA)-ABE product. And the predicted formula was C_46_H_76_N_6_O_11_ (ppm = 4.831) at *m/z* 889.5644. Using the HCD–MS/MS spectrum of the precursor ion [M + H]^+^ at *m/z* of 889.5644 (Figure 2B), -b and -y productions (Pathak et al., 2013) were assigned (Figure 2C), which enabled us to infer the sequence cyclo-[β-OHFA-Glu-Orn- Tyr-Thr -Ile]. The formula (C_46_H_76_N_6_O_11_) was consistent with the results of mass spectrometry (Table 1). The molecular mass ions at *m/z* 875.5488, 903.5801, and 917.5963 were found to be derived from a β-OHFA chain variant at *m/z* 889.5644 sharing the same peptide sequence (Figure 1D), based on the fragment ion profile obtained by high-resolution HCD–MS/MS. These lipopeptide precursor ions with a 14 Da (Yang et al., 2015) mass difference from *m/z* 889.5644 ion were assigned as new pentapeptide variants differing only in β-OHFA chain length.

**Table 1:** **Lipopeptides, retention times, m/z values of protonated forms and peptide sequences**.

**Approach of COM changes**	**Homologues of peptides**	**RT (min)**	**Molecular formula**	**Mass (m/z)**	**Peptide sequence**
Point mutation of donor ppsB (T7D)	A1	13.94	*n* = 12; C_45_H_74_N_6_O_11_	875.5498	β-OHFA-ABE
	A2	14.15	*n* = 13; C_46_H_76_N_6_O_11_	889.5688	Cyclo pentapeptide
	A3	14.15	*n* = 14; C_47_H_78_N_6_O_11_	903.5841	CH3(CH2)n1CHOHCH2CO-Glu-Orn-
	A4	14.15	*n* = 15; C_48_H_80_N_6_O_11_	917.6087	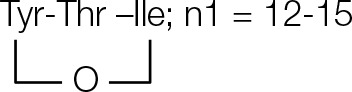
Deletion of donor ppsC	B1	13.07	*n* = 10; C_47_H_77_N_7_O_15_	980.5580	
	B2	13.15	*n* = 11; C_48_H_79_N_7_O_15_	994.5712	
	B3	13.20	*n* = 12; C_49_H_81_N_7_O_15_	1008.5873	β-OHFA-ABC
	B4	13.68	*n* = 13; C_50_H_83_N_7_O_15_	1022.6026	Linear hexapeptide
	B5	14.15	*n* = 14; C_51_H_85_N_7_O_15_	1036.6179	CH3(CH2)n2CHOHCH_2_CO-Glu-Orn-Tyr-Thr -Glu-Val; n2 = 10–16
	B6	14.15	*n* = 15; C_52_H_87_N_7_O_15_	1050.6338	
	B7	14.15	*n* = 16; C_53_H_89_N_7_O_15_	1064.6493	
the donor of ppsD replaced with ppsC	C1	15.63	*n* = 11; C_67_H_103_N_11_O_20_	1382.7556	β-OHFA-ABCD
	C2	15.63	*n* = 12; C_68_H_105_N_11_O_20_	1396.7715	Linear nonapeptide
	C3	15.98	*n* = 13; C_69_H_107_N_11_O_20_	1410.7876	CH_3_(CH_2_)_n3_CHOHCH_2_CO- Glu-Orn-Tyr-Thr-Glu-Val-Pro-Gln-Tyr; n3 = 11–13
the acceptor of ppsB replaced with ppsC	D1	12.15	n = 10; C_66_H_101_N_11_O_20_	1368.7387	β-OHFA-ABCD
	D2	12.33	n = 11; C_67_H_103_N_11_O_20_	1382.7517	Linear nonapeptide
	D3	13.15	n = 12; C_68_H_105_N_11_O_20_	1396.7667	CH_3_(CH_2_)_n4_CHOHCH_2_CO-
	D4	13.68	n = 13; C_69_H_107_N_11_O_20_	1410.7817	Glu-Orn-Tyr-Thr -Glu-Val -Pro-Gln-Tyr;n4 = 11–14
the donor of ppsC replaced with ppsD	E1	13.52	n = 12; C_53_H_88_N_8_O_16_	1093.6393	
	E2	13.68	n = 13; C_54_H_90_N_8_O_16_	1107.6598	β-OHFA-ABCE
	E3	14.15	n = 14; C_55_H_92_N_8_O_16_	1121.6753	Linear heptapeptide
	E4	14.69	n = 15; C_56_H_94_N_8_O_16_	1135.6871	CH_3_(CH_2_)_n5_CHOHCH_2_CO-Glu-Orn-
	E5	14.69	n = 16; C_57_H_96_N_8_O_16_	1149.7043	Tyr-Thr-Glu-Ala –Ile; n5 = 12–16
Deletion of donor ppsC and the acceptor of ppsC replaced with ppsB	F1	17.05	n = 12; C_64_H_98_N_10_O_16_	1263.7235	β-OHFA-ABDE
	F2	17.12	n = 13; C_65_H_100_N_10_O_16_	1277.7412	Cyclo octapeptide
	F3	17.68	n = 14; C_66_H_102_N_10_O_16_	1291.7504	CH_3_(CH_2_)_n6_CHOHCH_2_CO-Glu-Orn
	F4	18.06	n = 15; C_67_H_104_N_10_O_16_	1305.7682	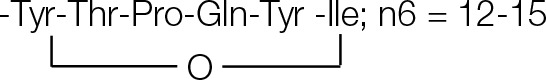

β-OHFA-ABE, The lipopeptides produced by NRPS complex assembly line ppsA, ppsB, and ppsE; β-OHFA-ABC, The lipopeptides produced by NRPS complex assembly line ppsA, ppsB, and ppsC; β-OHFA-ABCD, The lipopeptides produced by NRPS complex assembly line ppsA, ppsB, ppsC, and ppsD; β-OHFA-ABCE, The lipopeptides produced by NRPS complex assembly line ppsA, ppsB, ppsC, and ppsE; β-OHFA-ABDE, The lipopeptides produced by NRPS complex assembly line ppsA, ppsB, ppsD, and ppsE.

The T7D mutation resulted in a loss in the ability of the COM^D^ domain to form a productive complex with its native partner ${\text{COM}}_{\text{ppsB}}^{\text{A}}$ (the acceptor COM of ppsB). However, at the same time, ppsB point mutant gained the ability to interact with the ppsE. Meanwhile, a novel NRPS complex assembly line ppsA, ppsB and ppsE was formed. This indicated that the mutation of threonine in the donor COM domain of ppsB to aspartic acid was sufficient to transfer selectivity from the cognate acceptor COM toward the noncognate accepter COM of ppsD, and highlights the importance of the threonine residue at this position in protein-protein communication.

### Influence of ppsC donor deletion mutant on plipastatin synthesis

**Figure 3:**
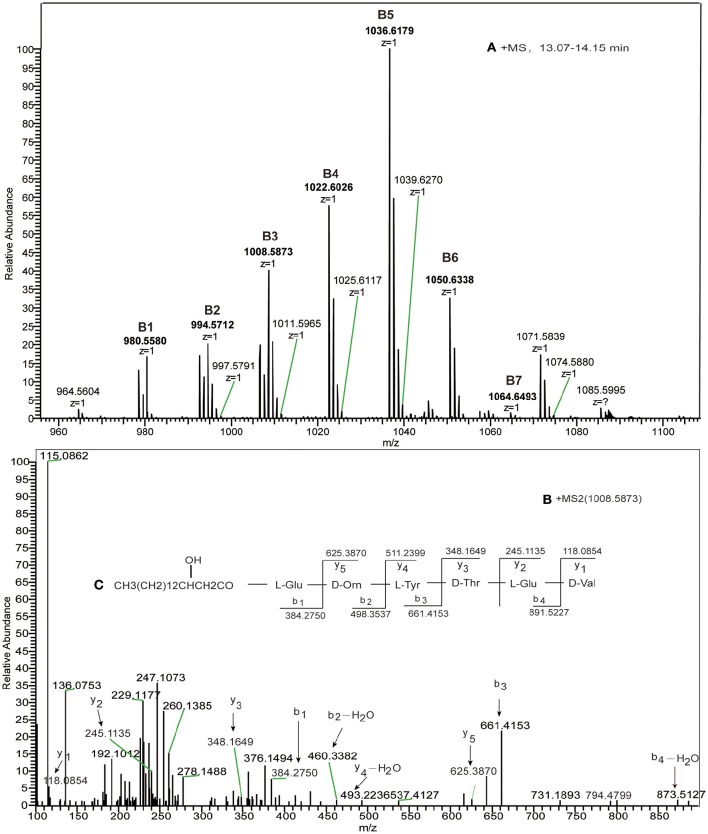
**(A)** The high-resolution ESI–MS of linear hexapeptide ions with RT in the range 13.07–14.15 min and **(B)** HCD–MS/MS of the precursor ion [M + H]^+^ at *m/z* 1008.5873, **(C)** which was confirmed as a novel hexapeptide consisting of Glu-Orn-Tyr-Thr-Glu-Val and a C_12_ β-OH fatty acid chain.

The deletion of ppsC donor on the ability to interact with the partner elongation module ppsD was investigated *in vivo* (Figure 1C). As shown in Table 1, the high-resolution ESI–MS revealed a series of charge 1 (*z* = 1) ions with *m/z* at 980.5560, 994.5712, 1008.5873, 1022.6026, 1036.6179, 1050.6338, and 1064.6493 (Figure 3A) that eluted with a retention time between 13.07 and 14.15 min. And the predicted formula was C_49_H_81_N_7_O_15_ (ppm = 0.673) at *m/z* 1008.5873. And the high-resolution HCD-MS/MS spectrum of the precursor ion (Figure 3B) was analyzed, and -b and -y ions were consistent with a linear [β-OHFA-Glu-Orn- Tyr-Thr-Glu-Val] peptide (Figure 3C), which lacked four amino acid residues (Pro, Gln, Tyr, and Ile) that are present in plipastatin. The formula of linear peptide was in accord with the MS spectra analysis. Furthermore, other molecular ions were clearly derived from this peptide moiety, and the 14 Da differences again indicated varying chain length of the β-OHFA (Figure 1D). The ${\text{COM}}_{\text{ppsC}}^{\text{D}}$ deletion largely resulted in a loss in the ability of the COM^*D*^ domain to form a productive complex with its native partner ${\text{COM}}_{\text{ppsC}}^{\text{A}}$. The deletion mutant was completely inactive in a product formation assay with ppsD, resulting in formation of the novel NRPS complex assembly line ppsA, ppsB, and ppsC.

### Influence of interchangeabilities in COM domain on plipastatin synthesis

Indeed, deletion of the donor module ppsC prevents plipastatin biosynthesis. However, the inability of mutant systems to synthesize plipastatin and the formation of novel products is due to either the loss of protein–protein interaction between donor and acceptor NRPS partners, or inactivity of donor modules causing by the deletion of the most C-terminal amino acid residues.

**Figure 4:**
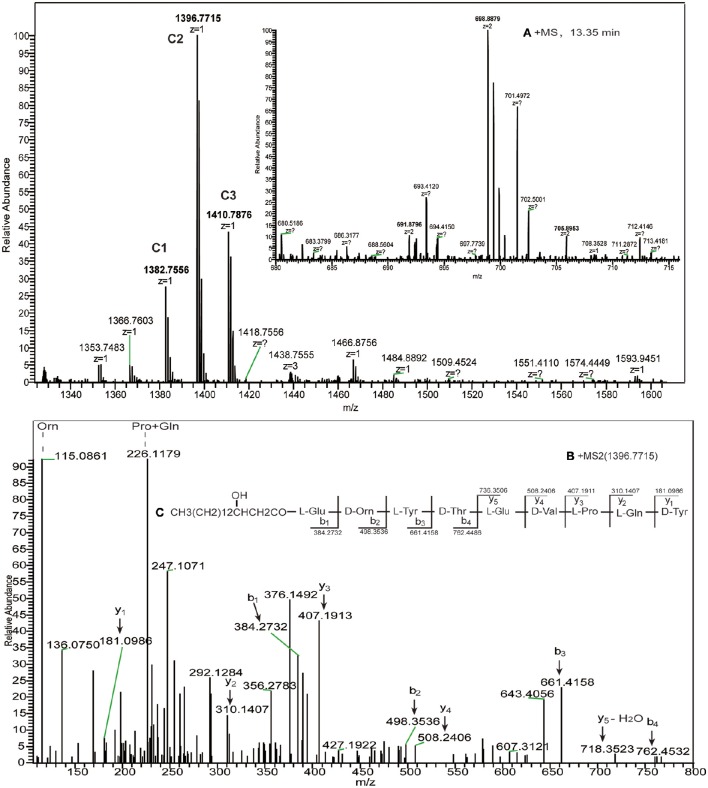
**(A)** The high-resolution ESI–MS of linear nonapeptide ions with RT 13.35 min and **(B)** HCD–MS/MS of the precursor ion [M + H]^+^ at *m/z* 1396.7715, **(C)** which was confirmed as a novel nonapeptide consisting of Glu-Orn-Tyr-Thr-Glu-Val-Pro- Gln-Tyr and a C_12_ β-OH fatty acid chain.

To challenge these potential explanations, crosstalk experiments were performed, which confirmed that members of the compatible pairs between ppsB and ppsC, ppsC and ppsD, as well as ppsD and ppsE were interchangeable *in vivo*. First, acceptor ppsC was initially swapped with ppsB, and donor ppsD was subsequently substituted with donor ppsC. Next, acceptor ppsB was mutated to acceptor ppsC, and donor ppsC was replaced by donor ppsD. Plipastatin was observed in the methanolic extract when acceptor ppsC was replaced by acceptor ppsB (data not shown), and the anticipated novel product was not formed. However, when ${\text{COM}}_{\text{ppsD}}^{\text{D}}$ was replaced by ${\text{COM}}_{\text{ppsC}}^{\text{D}}$, the high-resolution ESI–MS gave the charge 1 molecular mass ions of *m/z* 1382.7556, 1396.7715, and 1410.7876 (Figure 4A), which were assigned as hydrogen adducts of corresponding protonated novel nonapeptide ions. The charge 2 (*z* = 2) of the mass ions was 691.8796, 698.8879, and 705.8953, which verified the predicted formula was C_68_H_105_N_11_O_20_(ppm = 4.333). In order to confirm the assignments, the *m/z* 1396.7715 ion was subjected to the high-resolution HCD-MS/MS (Figure 4B), and the -y ion series at *m/z* 736.3506 (−H_2_O, 718.3523) → 508.2406 → 407.1911 → 310.1407 → 181.0986 was consistent with Glu-Val-Pro-Gln-Tyr from the N-terminus, while the -b ion series 762.4486 → 661.4158 → 498.3586 → 384.2732 suggested that the precursor ion possessed a Glu-Orn-Tyr-Thr at the C-terminus and a C12 β-OH fatty acid that lacked the Ile residue present in plipastatin, and the fragmentation pattern for the entire m/z 1396.7715 is consistent with MS/MS analysis (Figure 4C). Again, other molecular mass ions differing by 14 Da (−CH_2_−) were clearly derived from the same peptide sequence but with C11 and C13 β-OH fatty acids, respectively (Table 1, Figure 1D). This indicated that the donor of ppsD loss the ability of forming a productive complex with its native partner, and resulting the formation of the NRPS complex assembly line ppsA, ppsB, ppsC and ppsD.

**Figure 5:**
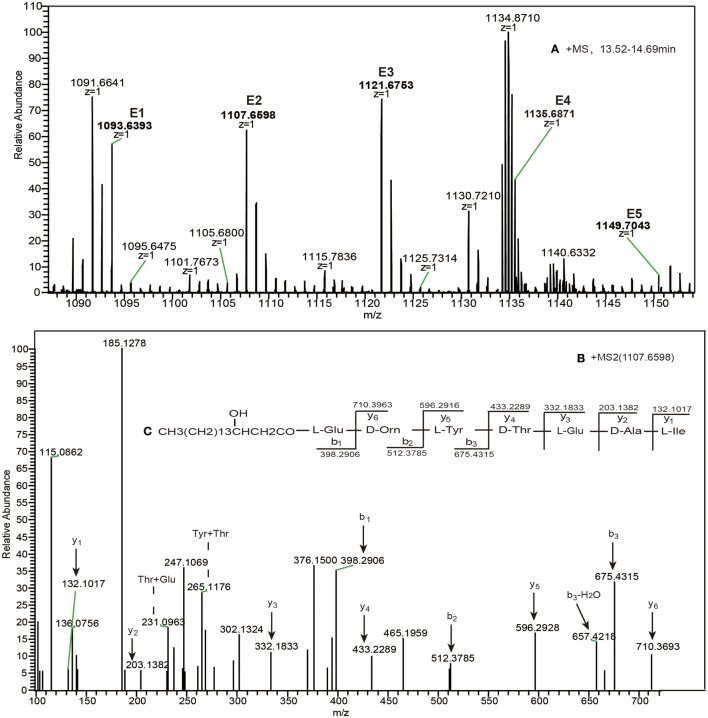
**(A)** The high-resolution ESI–MS of linear heptapeptide ions eluted with RT in the range 13.52–14.69 min and **(B)** HCD–MS/MS of the precursor ion [M + H]^+^ at *m/z* 1107.6598, **(C)** which was confirmed as a novel heptapeptide consisting of Glu-Orn-Tyr-Thr-Glu-Ala-Ile and a C_13_ β-OH fatty acid chain.

Subsequent mutation of acceptor ppsB to acceptor ppsC was predicted to result in a product with one further modification. The charge 1 (*z* = 1) molecular mass ions at *m/z* 1368.7387, 1382.7517, 1396.7667, and 1410.7817 and the charge 2 (*z* = 2) of the mass ions at *m/z* 684.8743, 691.8776, 698.8860, and 705.8932 were observed in the high-resolution ESI–MS spectrum (Figure S6A), which verified the predicted formula at *m/z* 1396.7667 was C_68_H_105_N_11_O_20_(ppm = 4.181). The high-resolution HCD-MS/MS fragmentation of the *m/z* 1396.7667 ion (Figure S6B) yielded a series of -b fragment ions at *m/z* 891.5045762.4468 → 661.4145 → 498.3531 → 384.2725 and -y fragment ions at m/z 736.3506 (−H_2_O, 718.3517) → 508.2404 → 407.1911 → 310.1393 → 181.0977 that were consistent with hydrogen adducts of a C12 β-OH FA and an nonapeptide with the sequence Glu-Orn-Tyr-Thr-Glu-Val- Pro-Gln- Tyr that is the same as that obtained with the modification described above, and is also lacking the Ile residue comparing the original product plipastatin. The result showed that the NRPS complex generated the assembly line ppsA, ppsB, ppsC, and ppsD. Finally, ${\text{COM}}_{\text{ppsB}}^{\text{D}}$ was replaced with ${\text{COM}}_{\text{ppsD}}^{\text{D}}$, this caused the disability of ${\text{COM}}_{\text{ppsB}}^{\text{D}}$ operon and generation of ${\text{COM}}_{\text{ppsB}}^{\text{D}}$ mutated to ${\text{COM}}_{\text{ppsD}}^{\text{D}}$ as expected. The high-resolution ESI–MS spectrum again included hydrogen adduct charge 1 (*z* = 1) mass ions differing by multiples of 14 Da that eluted between 13.52 and 14.69 min (Figure 5A). The formula of the [M + H]^+^ ion at *m/z* 1107.6598 was C_54_H_90_N_8_O_16_(ppm = 3.553) with the mass spectrum analysis. The high-resolution HCD-MS/MS fragmentation of *m/z* 1107.6598 ion eluting at 13.68 min resulted in -b and -y ion assignments (Figure 5C) consistent with a linear heptapeptide with a linear C13 β-OH FA and the sequence Glu-Orn-Tyr-Thr-Glu-Ala-Ile (Figure 5B). This sequence was produced by the NRPS complex assembly line ppsA, ppsB, ppsC, and ppsE, and lacks three amino acid residues (Pro, Gln and Tyr) that are present in plipastatin. Other linear heptapeptides identified in this study are summarized in Table 1, Figure 1D. Experiments unequivocally confirmed that the acceptor modules did not contribute to the formation of novel products, however, all tested donor modules were equally able to change the native system synthesizing plipastatin.

### Influence of combined deletion and substitution of COM domains on plipastatin synthesis

**Figure 6:**
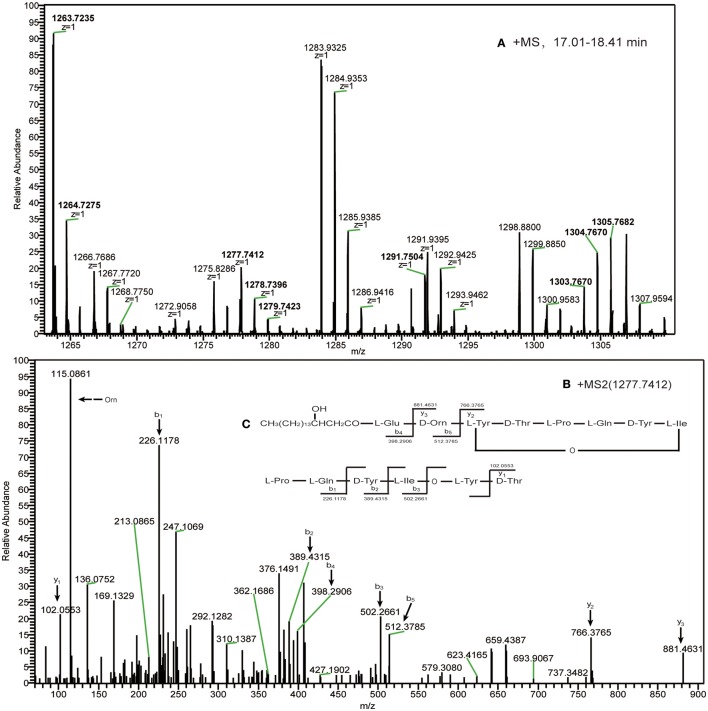
**(A)** The high-resolution ESI–MS of cyclic octapeptide ions with RT in the range 17.01–18.41 min and **(B)** HCD–MS/MS of the precursor ion [M + H]^+^ at *m/z* 1277.7412, **(C)** which was confirmed as a novel cyclic octapeptide consisting of Glu-Orn-Tyr-Thr-Pro-Gln-Tyr-Ile and a C_13_ β-OH fatty acid chain.

The second experiment demonstrated that the ppsC module could not couple with the next ppsD module following deletion of the ppsC donor. However, when the partner donor was present, altering the acceptor module had no effect on the formation of novel lipopeptides in the permutation experiment. We therefore decided to investigate whether the acceptor module retains the ability to interact when its partner donor module is deleted. The donor of ppsC was first deleted, and the acceptor of ppsC was then substituted with the acceptor of ppsB (Figure 1C). The high-resolution ESI–MS analysis of the resulting product gave a charge 1 (*z* = 1) molecular mass ion series of *m/z* 1263.7235, 1277.7412, 1291.7504, and 1305.7682 (Figure 6A) that corresponded with hydrogen adducts of ions derived from a novel cyclic octapeptide. The predicted formula was C_65_H_100_N_10_O_16_ (ppm = 1.132) at *m/z* 1277.7412. The high-resolution HCD-MS/MS fragmentation of the *m/z* 1277.7412 ion (Figure 6B) yielded a -y ion series at *m/z* 102.0553 → 766.3765 → 881.4631 from the N-terminus, and a -b ion series at *m/z* 226.1178 → 389.4315 → 502.2661 → 398.2906 → 512.3785 from the C-terminus, consistent with a cyclic Glu-Orn-Tyr- Thr-Pro-Gln-Tyr-Ile peptide and a C13 β-OH fatty acid lacking Glu and Ala/Val amino acid residues that are present in plipastatin and the fragmentation pattern is illustrated in Figure 6C. Again, other molecular mass ions (Table 1, Figure 1D) differing by 14 Da (−CH_2_−) were derived from the same peptide but with C11, C12, and C14 β-OH fatty acids. This indicated that a novel NRPS complex assembly line ppsA, ppsB, ppsD and ppsE directed the production of cyclic octapeptide.

Deletion of donor of ppsC largely abrogated the ability of the COM^D^ domain to form a productive complex with its native partner ${\text{COM}}_{\text{ppsC}}^{\text{A}}$, but formation of plipastatin was not affected when the acceptor of ppsC was substituted with the acceptor of ppsB. However, the including of both modifications mutant resulted in the ability of ppsB to interact with the ppsC acceptor (${\text{COM}}_{\text{ppsC}}^{\text{A}}$). Therefore, the acceptor module retained selectivity when its partner donor module was deleted.

### Antimicrobial activity

The antimicrobial activity of the linear lipopeptide products was tested against one Gram-positive bacteria, one Gram-negative bacteria and five fungi using a standard dilution approach. The lipopeptides exhibited antimicrobial activity against five of the fungal species at a contention of 31.25–125 μg/ml (Table 2). Interestingly, the novel lipopeptides inhibited both tested bacteria. *Penicillium notatum* exhibited the least resistance against linear heptapeptide and hexapeptide compared with all fungi, showing a MIC of 31.25 μg/ml, which was the value of the linear heptapeptide against *Aspergillus ochraceus.* The MIC values of all linear lipopeptides against *Escherichia coli* and *Staphylococcus aureus* were much higher than against the fungi, and the MIC values were, respectively, 250 and 500 μg/ml. The cyclic lipopeptides exhibited the inhibitory activity as shown in Figure S7.

**Table 2:** **Minimum inhibitory concentration (MIC) of lipopeptides against fungal species (μg/mL)**.

**Fungal species**	**Linear hexapeptide**	**Linear nonapeptide**	**Linear heptapeptide**
*Rhizopus stolonifer*	62.5	62.5	62.5
*Fusarium oxysporum*	62.5	62.5	62.5
*Aspergillus ochraceus*	62.5	62.5	31.25
*Penicillium notatum*	31.25	62.5	31.25
*Aspergillus flavus*	62.5	125	125
*Escherichia coli*	250	500	250
*Staphylococcus aureus*	250	500	500

## Discussion

Based on the characterization of NRPS systems, each of the large modular enzymes is deemed responsible for the incorporation of a monomeric precursor into a specific amino acid (Walsh, 2002; Schwarzer et al., 2003). In multienzymatic NRPS complexes, synthesis by most known NRP assembly lines requires appropriate communication between partner enzymes including A-PCP minimal modules (Schneider et al., 1998), C-A-PCP elongation modules (Mootz et al., 2002), A domains (Eppelmann et al., 2002), and translocation of the terminal, product-releasing Te domains (de Ferra et al., 1997). A recent study has examined the interaction between NRPS modules by introducing the photocrosslinking unnatural amino acidBpF (Chin et al., 2002) into aminimal, dimodular NRPS system. Further, the crosslinks were Photocrosslinked and mapped of by MS (Dehling et al., 2016). In addition, protein-protein communication is controlled by the interplay between linker (COM) domains that comprise only 20–30 or 15–25 amino acid residues, and that are located at the C and N termini between NRPS enzyme modular domains (Hahn and Stachelhaus, 2004). The selectivity provided by different sets of compatible COM^D^ and COM^A^ domains results in NRPS complexes that utilize defined biosynthetic templates and synthesize specific peptide products (Hahn and Stachelhaus, 2004, 2006).

In the present study, we investigated the selectivity of COM domains by direct reprogramming of the plipastatin biosynthetic complex, and achieved a biocombinatorial synthesis of new lipopeptides possessing significant antimicrobial activity. Novel lipopeptides sequences were identified by the structure of plipastatin (Deleu et al., 2008) coupled with HCD-MS/MS fragmentation (Pathak et al., 2013). In the first part of the study, the amino acid substitutions can be classified into four groups: (i) conservative exchanges between hydrophobic amino acids (A3L) and polar uncharged residues (S2T and G12S), (ii) substitution of a hydrophobic with a polar residue (W1K), (iii) exchange of a polar uncharged residue for a negatively charged residue (T7D), and (iv) substitution of a polar negatively charged residue with a polar positively charged (E9K) or uncharged (D10S) amino acid. The key residues that mediate the interaction between donor ppsB and acceptor ppsC COM domains were mutated, and the resultant NRPS system was able to form a novel assembly line (ppsA/ppsB/ppsE) that could synthesize lipopentapeptides. Indeed, a Thr-to-Asp mutation resulted in a series of homologous shortened pentapeptide products (Table 1). The previous research demonstrated that the selective communication is predominantly established by polar and/or electrostatic interactions in tyrocidine NRPS complex (Hahn and Stachelhaus, 2006). However, the substitution of a hydrophobic with a polar residue (W1K) did not change the selectivity. Therefore, the electrostatic interactions might play a key role in the selective communication. This approach can therefore be used to identify key residues that are important for maintaining (or preventing) the interaction between COM^D^ and COM^A^ in NRPSs. Deletion of the ppsC donor prevented selectivity of the acceptor of ppsC, and resulted in a novel assembly line (ppsA/ppsB/ppsC) that synthesized a lipohexapeptide. It was also demonstrated that the COM^A^ domain alone is insufficient for interacting with its upstream partner enzyme in the enzyme complex with specificity (Cheng et al., 2016). We next questioned whether both the donor and acceptor COM domains influence the selectivity by switching the acceptors of ppsC and ppsB. The differences between respective COM domain pairs were minimal (Figure S2) (Linne et al., 2003), and the mutation of acceptor COM domains impairs rather than halts product formation. In contrast, formation of plipastatin was not only impaired but completely abrogated when donor modules of COM domains were changed, and resulted in formation of new assembly lines (ppsA/ppsB/ppsC/ppsD and ppsA/ppsB/ppsC/ppsE) that synthesized novel lipononapeptide and lipoheptapeptide products. These results further demonstrated that donor COM domains influence interactions in the biosynthetic assembly line. Deletion of the ppsC donor ceased the interaction with its partner acceptor module, but when the partner acceptor module was mutated to the acceptor of ppsB, which facilitated the synthesis of a novel assembly line (ppsA/ppsB/ppsD/ppsE). Therefore, the acceptor COM domains retain selectivity when their partner donor is deleted. It was previously shown that when all donor and acceptor were replaced with the same (compatible) COM^D^ and COM^A^ domains in the surfactin biosynthetic complex, different assembly lines (SrfA-A/SrfA-B/SrfA-C and SrfA-A/SrfA-C) were formed that led to the synthesis of lipoheptapeptide and the lipotetrapeptide products (Chiocchini et al., 2006). A universal communication system for the plipastatin biosynthetic complex is therefore likely to result in additional novel lipopeptides.

Nonribosomal peptides synthetases (NRPSs) synthesis structurally diverse peptide-based natural products (Marahiel and Essen, 2009). Manipulation of their modular organization provides enormous potential for generating novel bioactive compounds (Miao et al., 2006; Butz et al., 2008; Doekel et al., 2008). Matching pairs of COM domains form protein–protein interactions (Linne et al., 2003; Hahn and Stachelhaus, 2006), and the present study demonstrated the decisive role of COM domain pairs in NRP biosynthetic complexes. The need for new antibiotics to fight the emergence of resistant pathogenic microorganisms is growing (Marr et al., 2006), and the novel lipopeptides proved to have good antimicrobial activity.

## Conclusions

Our results and experimental approaches pave the way for (1) identifying key residues in COM domains (2) characterizing the interactions between donor and acceptor COM domains, and (3) the preparation of novel lipopeptides possessing antimicrobial activity. Donor or acceptor modules could therefore influence the selectivity of NRPS systems. We demonstrated that (1) a single point mutation of a COM domain can alter the selectivity of the biosynthetic assembly line, (2) donor COM domains influence interactions in the biosynthetic assembly line, (3) mutation of acceptor COM domains impairs rather than halts product formation, (4) acceptor COM domains retain selectivity when their partner donor is deleted, and (5) the lipopeptides possess strong antifungal and some antibacterial activity. Our experimental approaches provide the theoretical basis for the production of the novel lipopeptides, which has potential for use in food preservation.

## Author contributions

XB conceived the project and revising it critically for important intellectual content, HL and LG designed the experiments, HL analyzed the data and wrote the manuscript. CD, JH, and ZM assisted in data analysis, XB, ZL, and CZ supervised the study.

### Conflict of interest statement

The authors declare that the research was conducted in the absence of any commercial or financial relationships that could be construed as a potential conflict of interest.
