# Supplementary material for: Biocombinatorial Synthesis of Novel Lipopeptides by COM Domain-Mediated Reprogramming of the Plipastatin NRPS Complex
Source: Front Microbiol. 2016 Nov 17;7:1801. doi: 10.3389/fmicb.2016.01801 (PMC5112269; doi:10.3389/fmicb.2016.01801)
Supplement: Supplementary file 1 [file Presentation1.ZIP › supplementary material/Linear heptapeptide.pdf]

CERTIFICATION OF ANALYSIS

Product Information:

Product Name	
Cat. NO.	816908
Size:	2mg*1
Sequence:	3-HYDROXYHEXADECANOIC ACID-Glu-(D-Orn)-Tyr-(D-allo-Thr)-Glu-(D-Val)-Ile
Molecular Weight:	1121.26
Storage:	-20℃

Analysis Summary:

Test Items	Standard	Result
HPLC Trace:	N/A	N/A
Mass Spectrometry:	Consistent	Consistent
Appearance:	White powder	Consistent

Caution:
For laboratory or further manufacturing use only. Not for household or any human being related utilize. If there is any further question, please contact **KareBay^{TM} BioChem** at:
Tel: 732-823-1545
E-mail: support@karebaybio.com.

816908 HPLC Analysis Report

Sample: 3-HYDROXYHEXADECANOIC ACID-Glu-(D-Orn)-Tyr-(D-allo-Thr)-Glu-(D-Val)-Ile
Sample ID:816908
Buffer A:0.1% TFA in 100% water(v/v)
Buffer B:0.1% TFA in 80% acetonitrile+ 20% water(v/v)
Gradient:40-100% Buffer B in 20min + 100% Buffer B in 10min
Flow:1ml/min Wavelength:220nm
Column: Agilent Pursuit 5um C18 4.6*250mm

Chromatogram

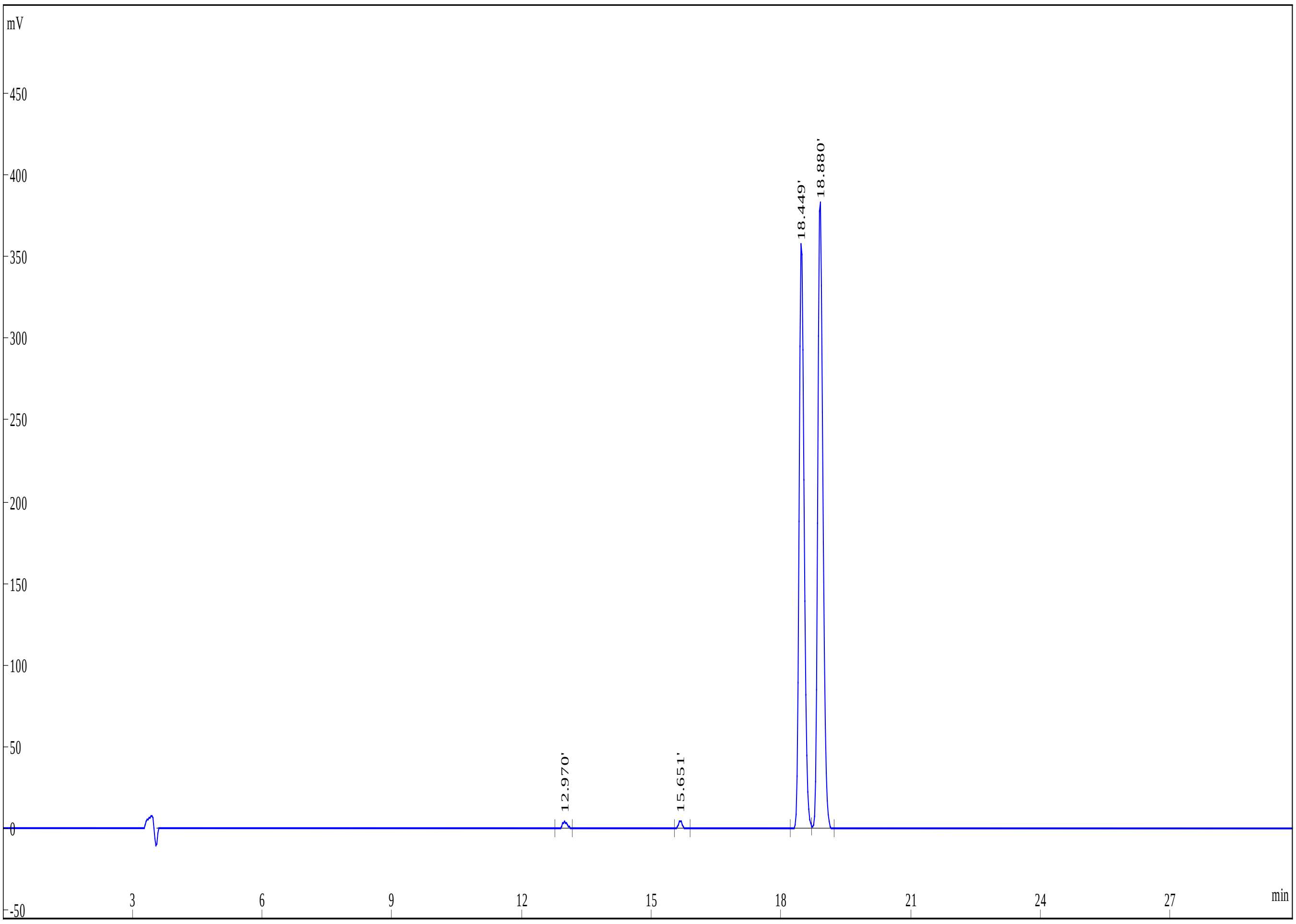

Signal	Peak#	RT(min)	Area%	Area(mAU*s)	Height(mAU)
1		12.970	0.5874	36298	4505
2		15.651	0.5551	34301	5153
3		18.449	46.87	2895899	365520
4		18.880	51.99	3212560	391606
Total			100	6179058	766784

MS Spectrum

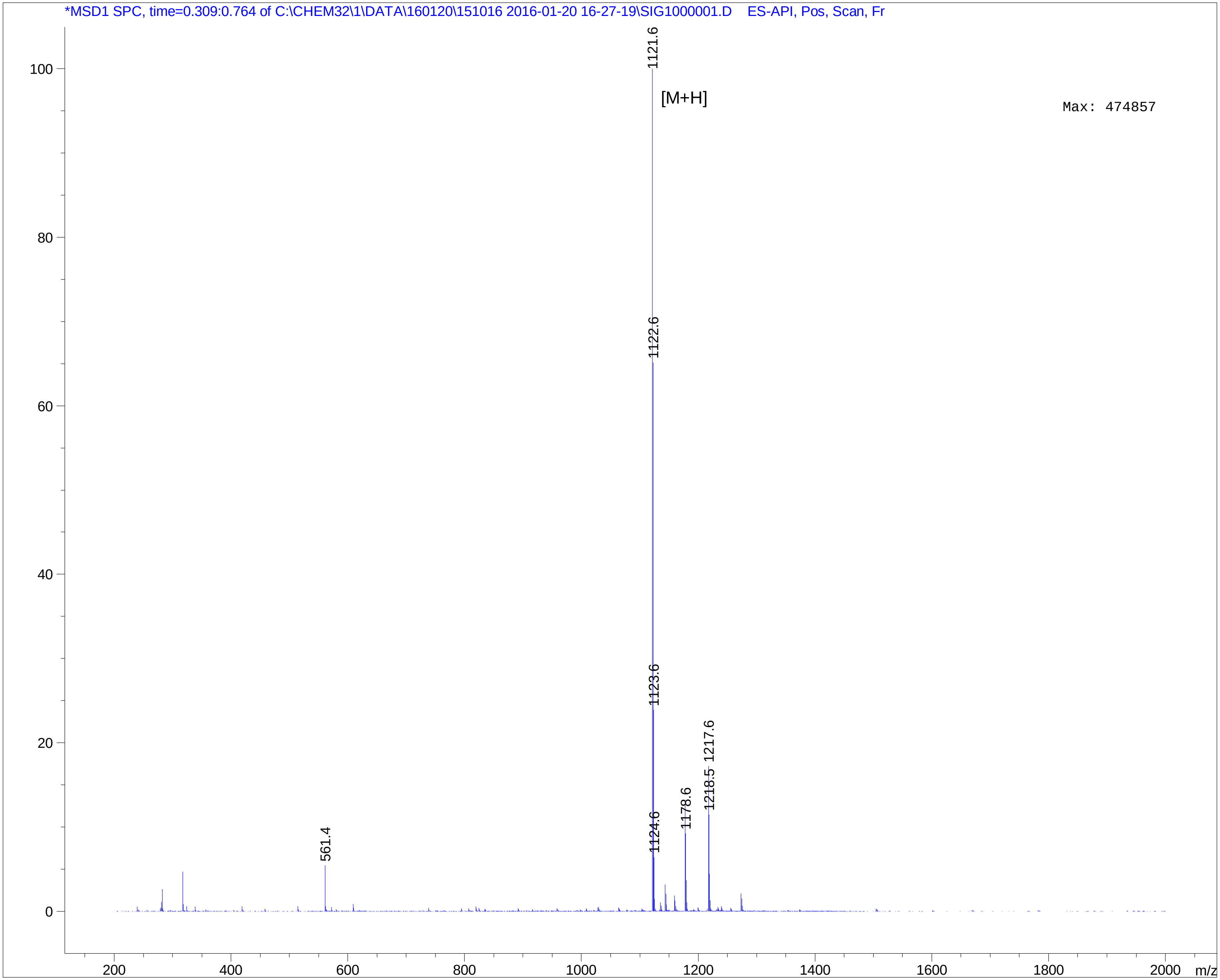
*MSD1 SPC, time=0.309:0.764 of C:\CHEM32\1\DATA\160120\151016 2016-01-20 16-27-19\SIG1000001.D ES-API, Pos, Scan, Fr
[M+H]
Max: 474857
1121.6
1122.6
1123.6
1124.6
1178.6
1217.6
1218.5
561.4
0
20
40
60
80
100
200
400
600
800
1000
1200
1400
1600
1800
2000
m/z
