# Supplementary material for: Biocombinatorial Synthesis of Novel Lipopeptides by COM Domain-Mediated Reprogramming of the Plipastatin NRPS Complex
Source: Front Microbiol. 2016 Nov 17;7:1801. doi: 10.3389/fmicb.2016.01801 (PMC5112269; doi:10.3389/fmicb.2016.01801)
Supplement: Supplementary file 1 [file Presentation1.ZIP › supplementary material/Linear hexapeptide.pdf]

CERTIFICATION OF ANALYSIS

Product Information:

**Product Name**	
Cat. NO.	816907
Size:	2mg*1
Sequence:	3-HYDROXYHEXADECANOIC ACID-Glu-(D-Orn)-Tyr-(D-allo-Thr)-Glu-(D-Val)
Molecular Weight:	1008.26
Storage:	-20℃

Analysis Summary:

**Test Items**	**Standard**	**Result**
HPLC Trace:	N/A	N/A
Mass Spectrometry:	Consistent	Consistent
Appearance:	White powder	Consistent

Caution:
For laboratory or further manufacturing use only. Not for household or any human being related utilize. If there is any further question, please contact **KareBay^{TM} BioChem** at:
Tel: 732-823-1545
E-mail: support@karebaybio.com.

816907 HPLC Analysis Report

Sample: 3-HYDROXYHEXADECANOIC ACID-Glu-(D-Orn)-Tyr-(D-allo-Thr)-Glu-(D-Val)

Sample ID: 816907

Buffer A:0.1% TFA in 100% water(v/v)

Buffer B:0.1% TFA in 80% acetonitrile + 20% water(v/v)

Gradient:40-100% Buffer B in 20min + 100% Buffer B in 10min

Flow:1ml/min Wavelength:220nm

Column: Agilent Pursuit 5um C18 4.6*250mm

Chromatogram

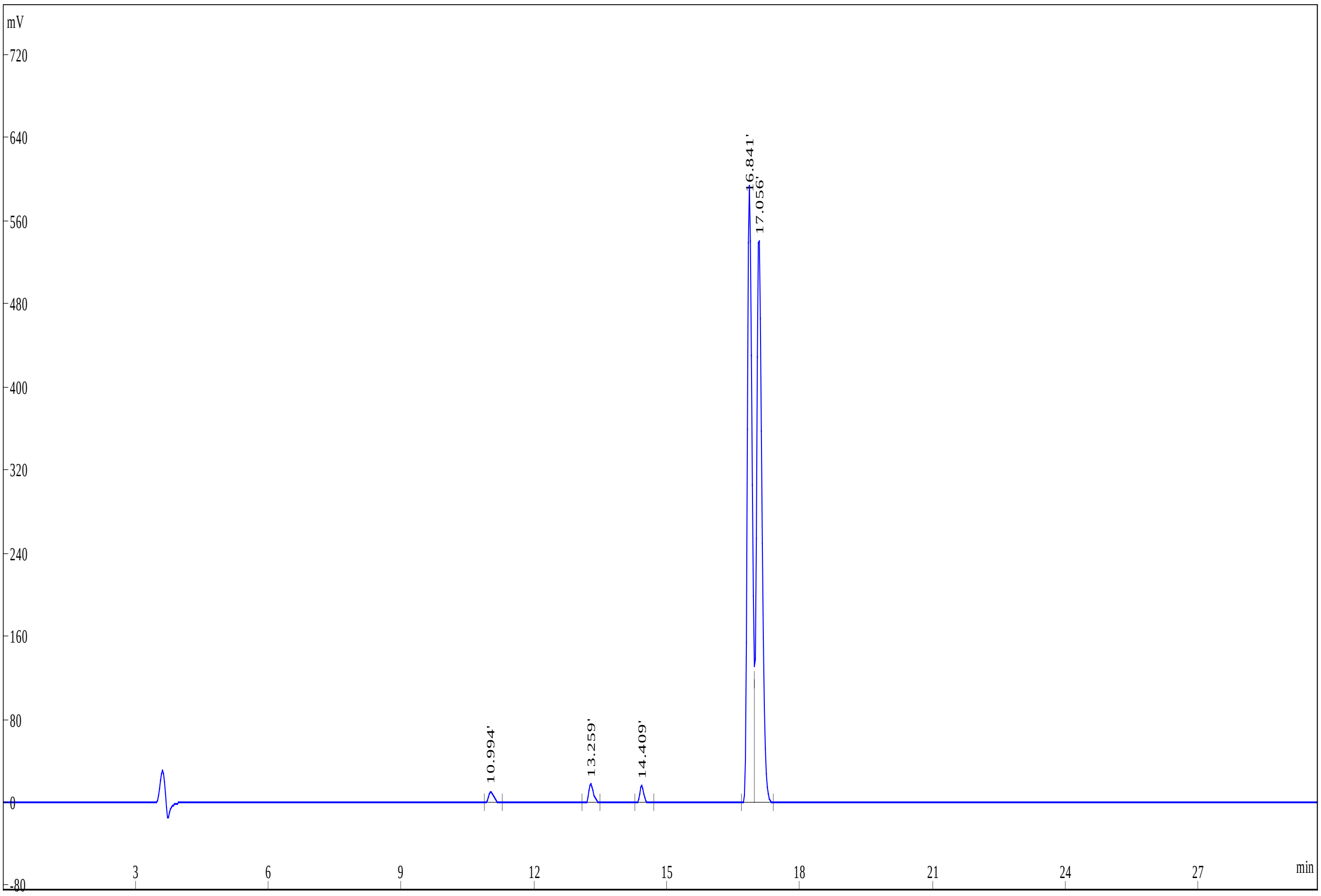

Signal	Peak#	RT(min)	Area%	Area(mAU*s)	Height(mAU)
1		10.994	0.9474	88077	11353
2		13.259	1.428	132715	18047
3		14.409	1.142	106173	16854
4		16.841	47.97	4459691	598637
5		17.056	48.51	4510109	556378
Total			100	9296765	1201269

Address: 11 Deer Park Drive, Suite 204, Monmouth Junction, NJ 08852
Telephone: 732-823-1545 **Email:** service@karebaybio.com **Website:** www.karebaybio.com

MS Spectrum

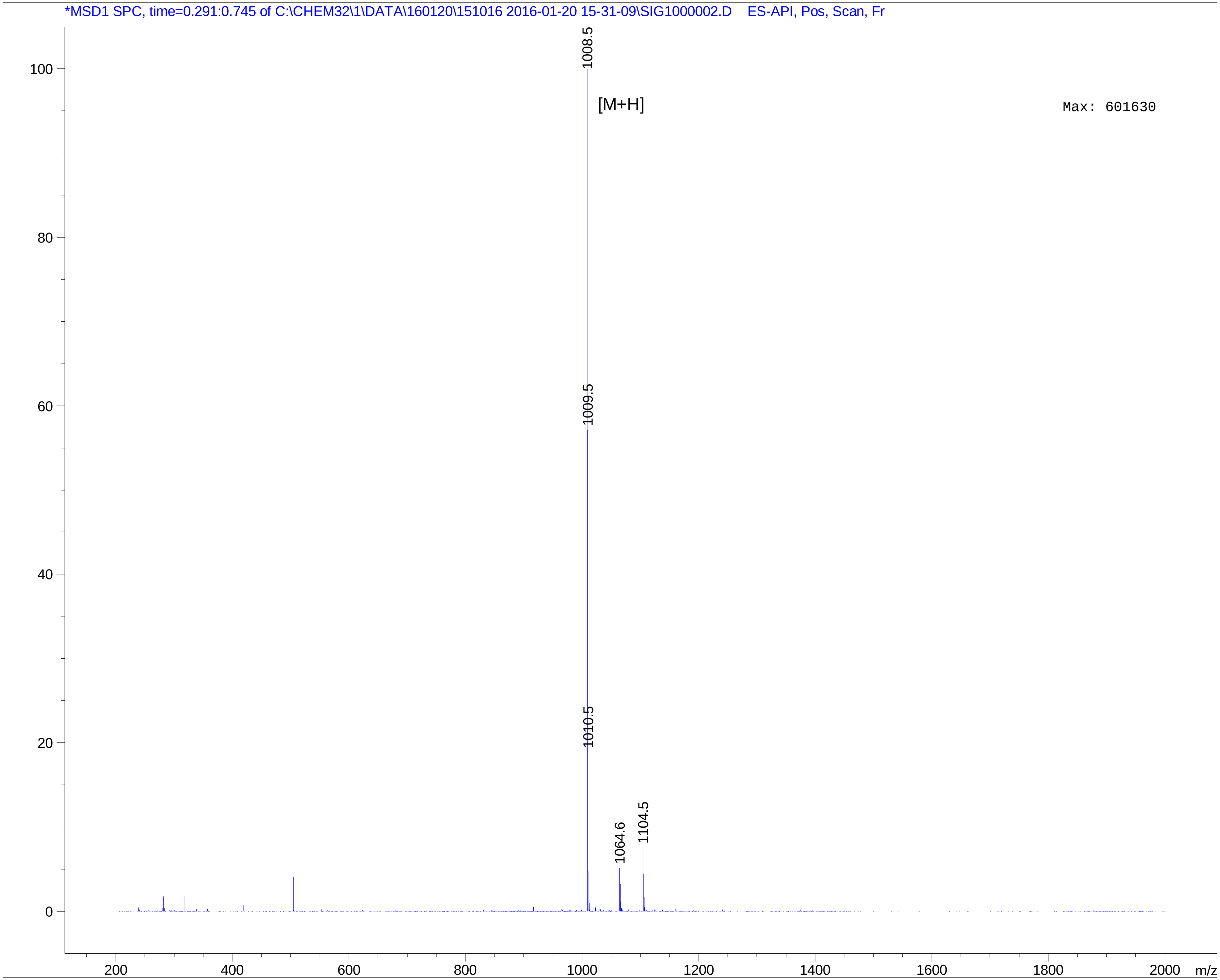
*MSD1 SPC, time=0.291:0.745 of C:\CHEM32\1\DATA\160120\151016 2016-01-20 15-31-09\SIG1000002.D ES-API, Pos, Scan, Fr
1008.5
[M+H]
Max: 601630
1009.5
1010.5
1064.6
1104.5
m/z
