# Supplementary material for: Biocombinatorial Synthesis of Novel Lipopeptides by COM Domain-Mediated Reprogramming of the Plipastatin NRPS Complex
Source: Front Microbiol. 2016 Nov 17;7:1801. doi: 10.3389/fmicb.2016.01801 (PMC5112269; doi:10.3389/fmicb.2016.01801)
Supplement: Supplementary file 1 [file Presentation1.ZIP › supplementary material/Linear nonapeptide.pdf]

# CERTIFICATION OF ANALYSIS

## Product Information:

| Product Name | |
|---|---|
| Cat. NO. | 816718 |
| Size: | 2mg*1 |
| Sequence: | 3-HYDROXYHEXADECANOIC ACID-Glu-(D-Orn)-Tyr-(D-allo-Thr)-Glu-(D-Val)-Pro-Gln-(D-Tyr) |
| Molecular Weight: | 1396.69 |
| Storage: | -20℃ |

## Analysis Summary:

| Test Items | Standard | Result |
|---|---|---|
| HPLC Trace: | N/A | N/A |
| Mass Spectrometry: | Consistent | Consistent |
| Appearance: | White powder | Consistent |

**Caution:**
For laboratory or further manufacturing use only. Not for household or any human being related utilize. If there is any further question, please contact **KareBay[TM] BioChem** at:
Tel: 732-823-1545
E-mail: support@karebaybio.com.

# 816718 HPLC Analysis Report

Sample: 3-HYDROXYHEXADECANOIC ACID-Glu-(D-Orn)-Tyr-(D-allo-Thr)-Glu-(D-Val)-Pro-Gln-(D-Tyr)
Sample ID:816718
Buffer A:0.1% TFA in 100% water(v/v)
Buffer B:0.1% TFA in 80% acetonitrile+ 20% water(v/v)
Gradient:40-100% Buffer B in 20min + 100% Buffer B in 10min
Flow:1ml/min Wavelength:220nm
Column:Venusil C18 5um4.6*250mm

Chromatogram

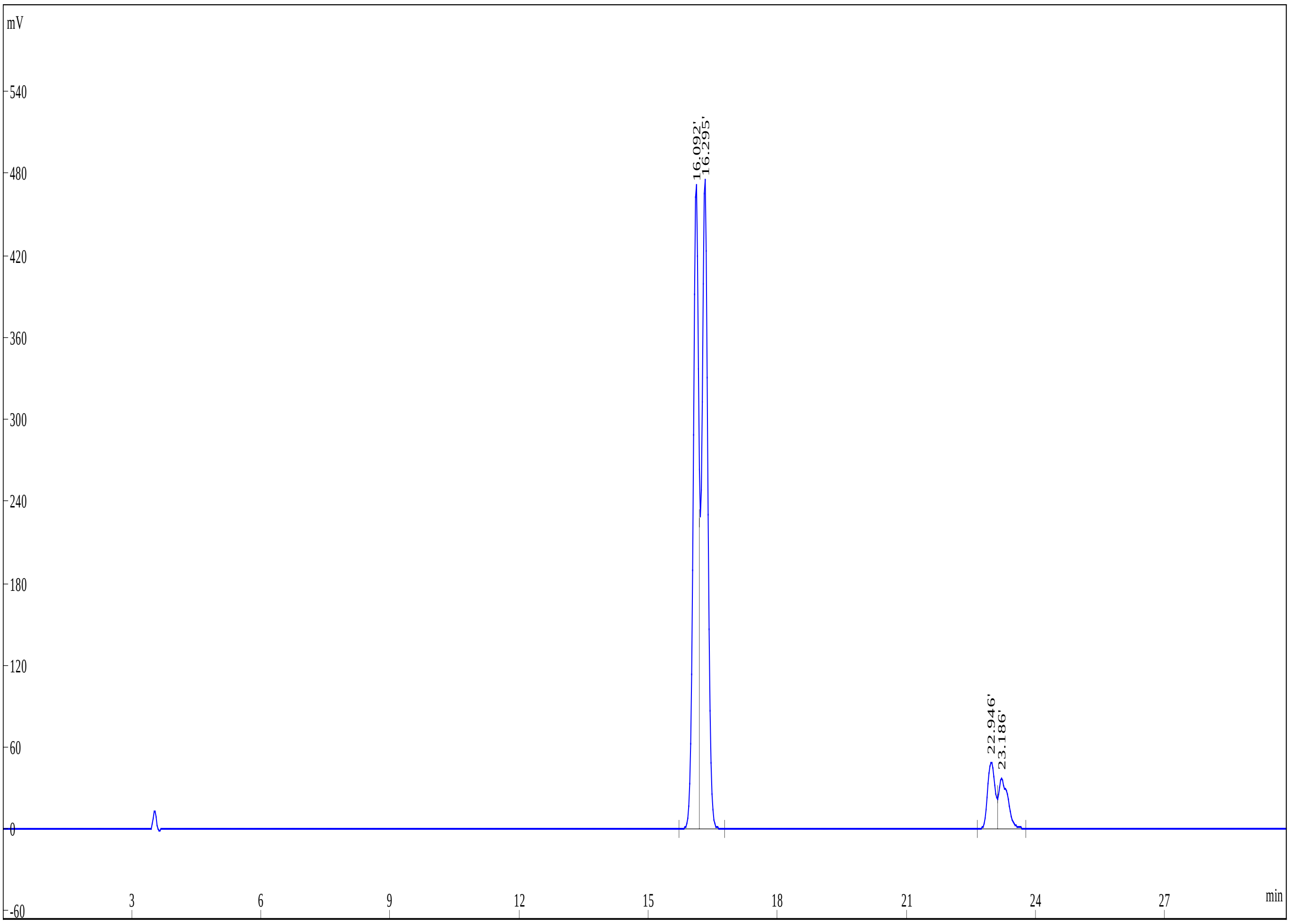


| Signal | Peak# | RT(min) | Area% | Area(mAU*s) | Height(mAU) |
|---|---|---|---|---|---|
| 1 | | 16.092 | 43.95 | 4394869 | 478851 |
| 2 | | 16.295 | 44.27 | 4426820 | 482053 |
| 3 | | 22.946 | 6.451 | 645065 | 49215 |
| 4 | | 23.186 | 5.326 | 532561 | 37544 |
| Total | | | 100 | 9999315 | 1047663 |

MS Spectrum

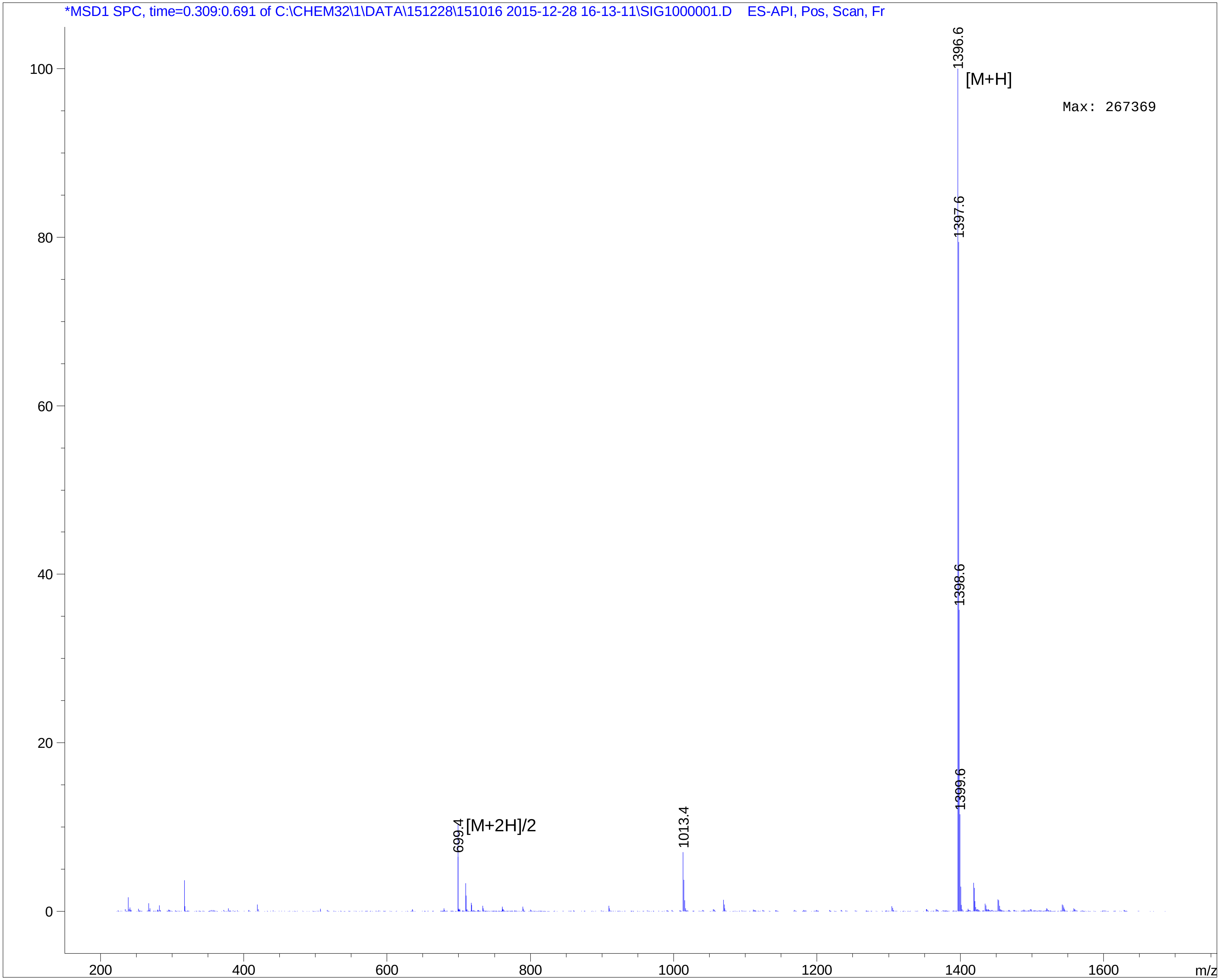
*MSD1 SPC, time=0.309:0.691 of C:\CHEM32\1\DATA\151228\151016 2015-12-28 16-13-11\SIG1000001.D ES-API, Pos, Scan, Fr
Max: 267369
1396.6
[M+H]
1397.6
1398.6
1399.6
699.4
[M+2H]/2
1013.4
m/z
