# Supplementary material for: Biocombinatorial Synthesis of Novel Lipopeptides by COM Domain-Mediated Reprogramming of the Plipastatin NRPS Complex
Source: Front Microbiol. 2016 Nov 17;7:1801. doi: 10.3389/fmicb.2016.01801 (PMC5112269; doi:10.3389/fmicb.2016.01801)
Supplement: Supplementary file 1 [file Presentation1.ZIP › supplementary material/supplementary material.docx]

**Biocombinatorial Synthesis of Novel Lipopeptides by COM Domain-Mediated Reprogramming of the Plipastatin NRPS Complex**

Hongxia Liu^1^, Ling Gao^1^, Jinzhi Han^1^, Zhi Ma^1^, Zhaoxin Lu^1^, Chen Dai^2^, Chong Zhang^1^ and Xiaomei Bie*^1^

^1^College of Food Science and Technology, Nanjing Agricultural University, Key Laboratory of Food Processing and Quality Control, Ministry of Agriculture of China, 1 Weigang Nanjing 210095, P.R. China

^2^College of Life Science, Nanjing Agricultural University, Ministry of Agriculture of China, 1 Weigang Nanjing 210095, P.R. China

***Corresponding Author:**

Xiaomei Bie

Nanjing Agricultural University

College of Food Science and Technology

1 Weigang

Nanjing, China 210095

[bxm43@jiau.edu.cn](mailto:bxm43@jiau.edu.cn)

The number of pages, figures, and tables were 7, 5, and 3.

**Table S1.** **Strains and plasmids.**

| Strain or plasmid | Characteristics | Source or Reference |
| --- | --- | --- |
| DH5α | FΦ80dlacZ ΔM12 minirecA1 | TaKaRa Bio Inc. |
| *B.Subtilis* |  |  |
| PB2 | Domesticated model, 168 derivative, | Laboratory stock |
| PB2-L  PB2-LM | PB2 derivative producing lipopeptides, polyketides, and surfactin, *sfp*+, *degQ*+, Cmr  PB2-L derivative with ppsB-COM^D^ point mutation, *sfp*+, *degQ*+ | The modified  This study |
| PB2-LK | PB2-L derivative without ppsC-COM^D^, *sfp*+, *degQ*+ | This study |
| PB2-LP1 | PB2-L derivative with the ppsD-COM^A^ replaced by ppsC-COM^A^ | This study |
| PB2-LP2  PB2-LP3  PB2-LP4  PB2-LKP1 | PB2-LP1 derivative with the ppsD-COM^D^ replaced by ppsC-COM^D^  PB2-LP2 derivative with the ppsB-COM^A^ replaced by ppsC-COM^A^  PB2-LP3 derivative with the ppsC-COM^D^ replaced by ppsD-COM^D^  PB2-LK1 derivative with the ppsC-COM^A^ replaced by ppsB-COM^A^ | This study  This study  This study  This study |
| Plasmids |  | This study |
| pMD 19-T | Cloning vector, Amp^r^ | TaKaRa Bio Inc |
| pMD1 | pMD19-T carrying the upstream A1, Amp^r^ | This study |
| pMD2  pMD3 | pMD19-T carrying the downstream A2, Amp^r^  pMD19-T carrying the upstream B1, downstream B2 and target fragment , Amp^r^ | This study  This study |
| pks2 | Temperature-sensitive vector, Erm^r^,Kan^r^ | This study |
| pks2A1A2 | Temperature-sensitive vector carrying A1 and A2 , Erm^r^,Kan^r^ | This study |
| Pks2B1B2 | Temperature-sensitive vector carrying B1, B2  and target fragment , Erm^r^,Kan^r^ | This study |

Cmr = chloramphenicol, Ampr = ampicillin, Ermr = erythromycin, Kanr = kanamycin resistance.

**Table S2. Oligonucleotide primers used in this study.**

| Primer name | Sequence (5′–3′) |
| --- | --- |
| Construction of  mutation strain  ppsB-F  ppsB-R  M1-F  M1-R  M2-F  M2-R  M3-F  M3-R  M4-F  M4-R  M5-F  M5-R  M6-F  M6-R  M7-F  M7-R  M8-F  M8-R  Construction of knockout strain | GCATGCCTGGGATGAGTGCGAGAA  GTCGACGGAGGCAAATCAGGAAGC  ATCTGATTTCGATGATGAAGACTTAACGCTTGATGAATTG  GTCTTCATCATCGAATCAGATTGCGCTCCACTCTTGGTTTTC  AAACCAAGAGAAGAGCGCATCTGATTTCACTGATGAAGAC  AGATGCGCTCTTCTCTTGGTTTTCTTTGCCGGTACAATGCTC  CAAGAGTGGACCGCATCTGATTTCACTGATGAAGACTTAACG  ATCAGATGCGGTCCACTCTTGGTTTTCTTTGCCGGTACAATG  AGAGTGGAGCTTATCTGATTTCACTGATGAAGACTTAACGCTTG  GAAATCAGATAAGCTCCACTCTTGGTTTTCTTTGCCGGTACAA  ATCTGATTTCGATGATGAAGACTTAACGCTTGATGAATTG  GTCTTCATCATCGAATCAGATTGCGCTCCACTCTTGGTTTTC  TTTCACTGATAAAGACTTAACGCTTGATGAATTGAGTGAG  GTTAAGTCTTTATCAGTGAAATCAGATGCGCTCCACTCTTG  CACTGATGAATCCTTAACGCTTGATGAATTGAGTGAGATC  AAGCGTTAAGGATTCATCAGTGAAATCAGATGCGCTCCAC  TGAGATCATGTCAGCCGTCAACAAACTATAGGAGAGGAG  GTTGACGGCTGACATGATCTCACTCAATTCATCAAGCGTTAA |
| K-up-F | ATCGATACCGGAGATTACTTGTTCCT ( *Cla* I) |
| K-up-R | GTCGACGTCTTTTCTGCTTTCTGTTG ( *Sal* I) |
| K-down-F | GTCGACAAAATCGTGACAGGAGACAT (*Sal* I) |
| K-down-R | GGTACCAGAATATGATCAATCAGCCC (*Kpn* I) |
| Construction of permutation strains |  |
| P1-F | ATGCCGCAGCAACCTGAAattcaggatatttatccgctgtcttaca |
| P1-R | TGAATTTCAGGTTGCTGCGGCATGTTCATGTCTCCTGTCACGATTTT |
| P2-up-F | GTCGACctgctcgcttctgtcat (*Sal* I) |
| P2-up-R  P2-down1-F  P2-down2-F | tgctgaaatcgctcagcgtcttttctgtttcgc  gacagcatatccagcttggtggaagaattgtaatgaatccgtgaag  CAGtcaatcattaacggcagaggacttggacagcatatccagc |
| P2-down3-F | cgaaacagaaaagacgctgagcgatttcagcagtcaatcatta |
| P2-down-R | GGTACCgtctcggtttgtcagccatg (*Kpn* I) |
| P3-F | ATTCACTGATGacaaaagcgaattcaATACAGGATATATACCCTTTGTCA |
| P3-R | ATATCCTGTATtgaattcgcttttgtCATCAGTGAATCTC CTCTCCTAT |
| P4-up-F | GTCGACcgtcttgctgcaaaacgtca (*Sal* I) |
| P4-up-R  P4-down1-F  P4-down2-F | CATCAAAATCACTGATTGTCTTTTCTGCTTTCTG  aagagatcgctgatatgctcagttttcactagaaaatcgtgacag  atgatcaagaactgaccgaggacgccctgcaagagatcgctgat |
| P4-down3-F | cagaaagcagaaaagacaatcagtgattttgatgatcaagaact |
| P4-down-R | GGTACCcaataagaaaccgcctgctc (*Kpn* I) |

Restriction sites are underlined

**Table S3. COM domain changes and resulting peptides.**

| COM domain change | Resultant peptide |
| --- | --- |
| COM^D^_ppsB_(W1K) point mutation | Cyclic plipastatin |
| COMDppsB(S2T) point mutation | Cyclic plipastatin |
| COM^D^_ppsB_(A3L) point mutation | Cyclic plipastatin |
| COM^D^_ppsB_(T7D) point mutation | Cyclic pentapeptide |
| COM^D^_ppsB_(E9K) point mutation | Cyclic plipastatin |
| COM^D^_ppsB_(D10S) point mutation | Cyclic plipastatin |
| COM^A^_ppsB_(G12S) point mutation | Cyclic plipastatin |
| COM^D^_ppsC_ deletion | Linear hexapeptide |
| COM^A^_ppsC_→COM^A^_ppsB_ | Cyclic plipastatin |
| COM^D^_ppsD_→COM^D^_ppsC_ | Linear nonapeptide |
| COM^A^_ppsB_→COM^A^_ppsC_ | Linear nonapeptide |


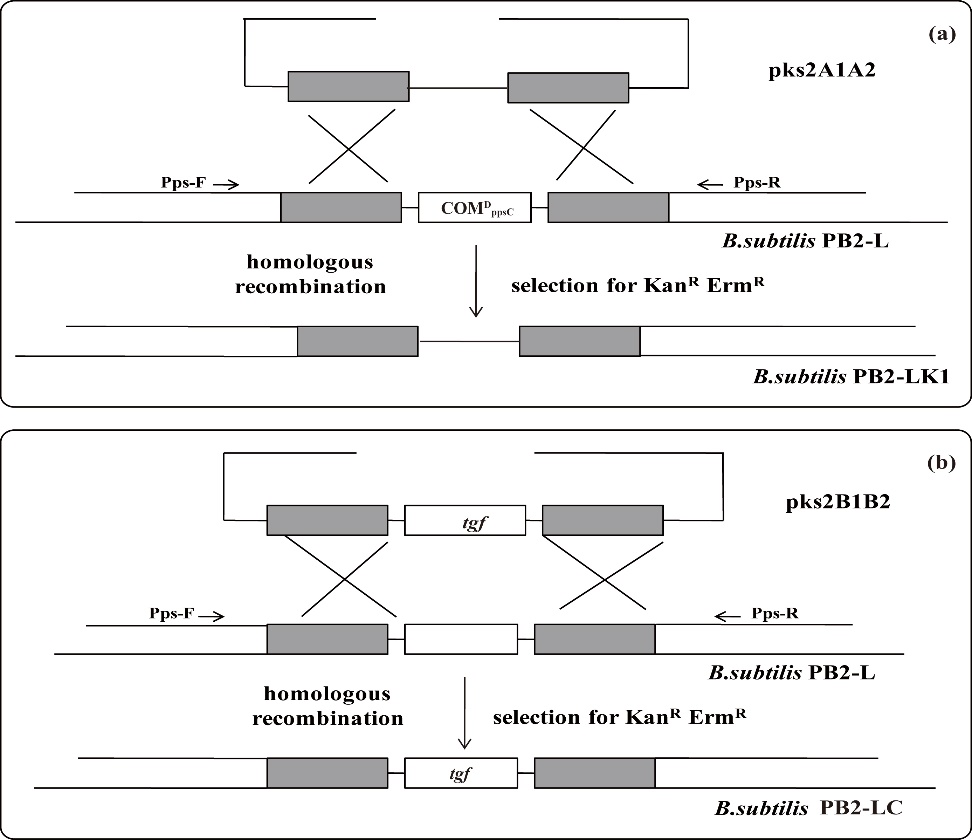


**Fig. S1** Linker domain changes. (a) Transformation of *Bacillus subtilis* PB2-L with pks2A1A2 led to homologous recombination and knockout of COM^D^_ppsC_. (b) Transformation of *B. subtilis* PB2-L with pks2B1B2 led to homologous recombination; *tgf* represents the target gene fragments of point mutations and substitutions.


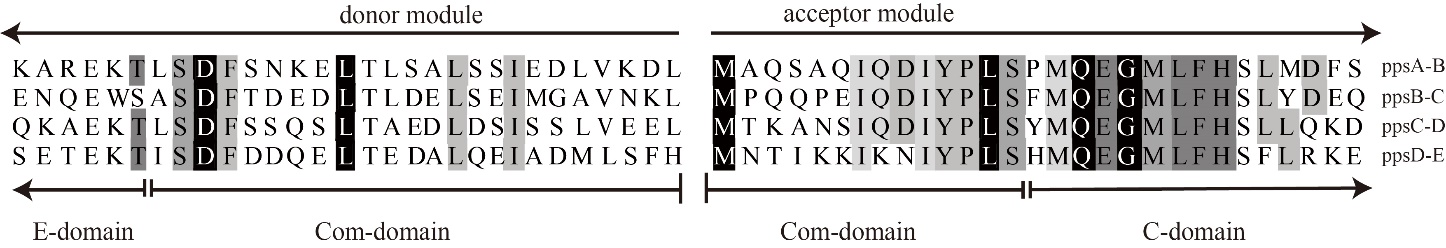


**Fig. S2** Sequence comparison of proposed donor and acceptor modules derived from the plipastatin biosynthetic system.


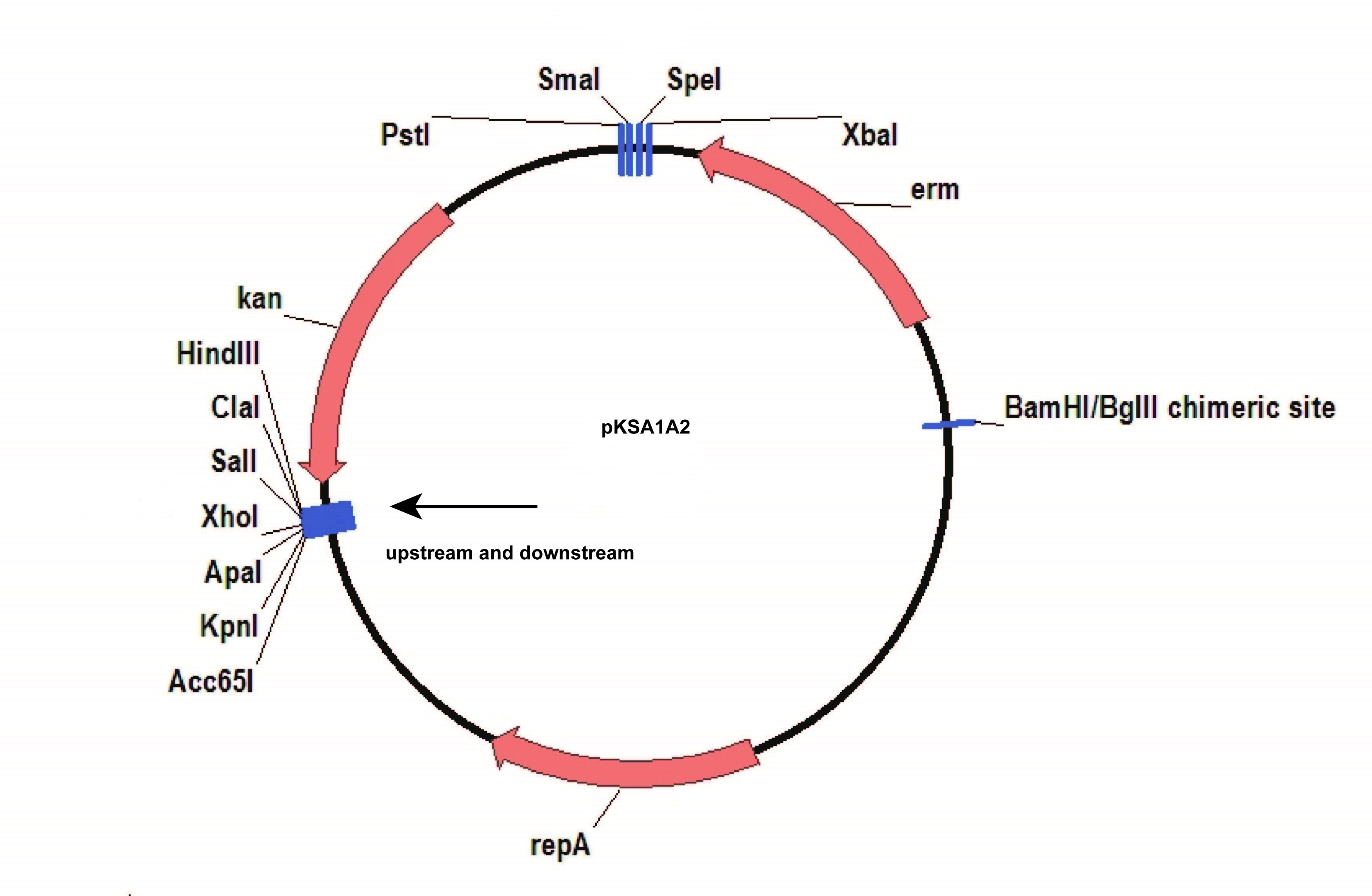


**Fig. S3** Map of plasmids used to construct the deletion mutant.


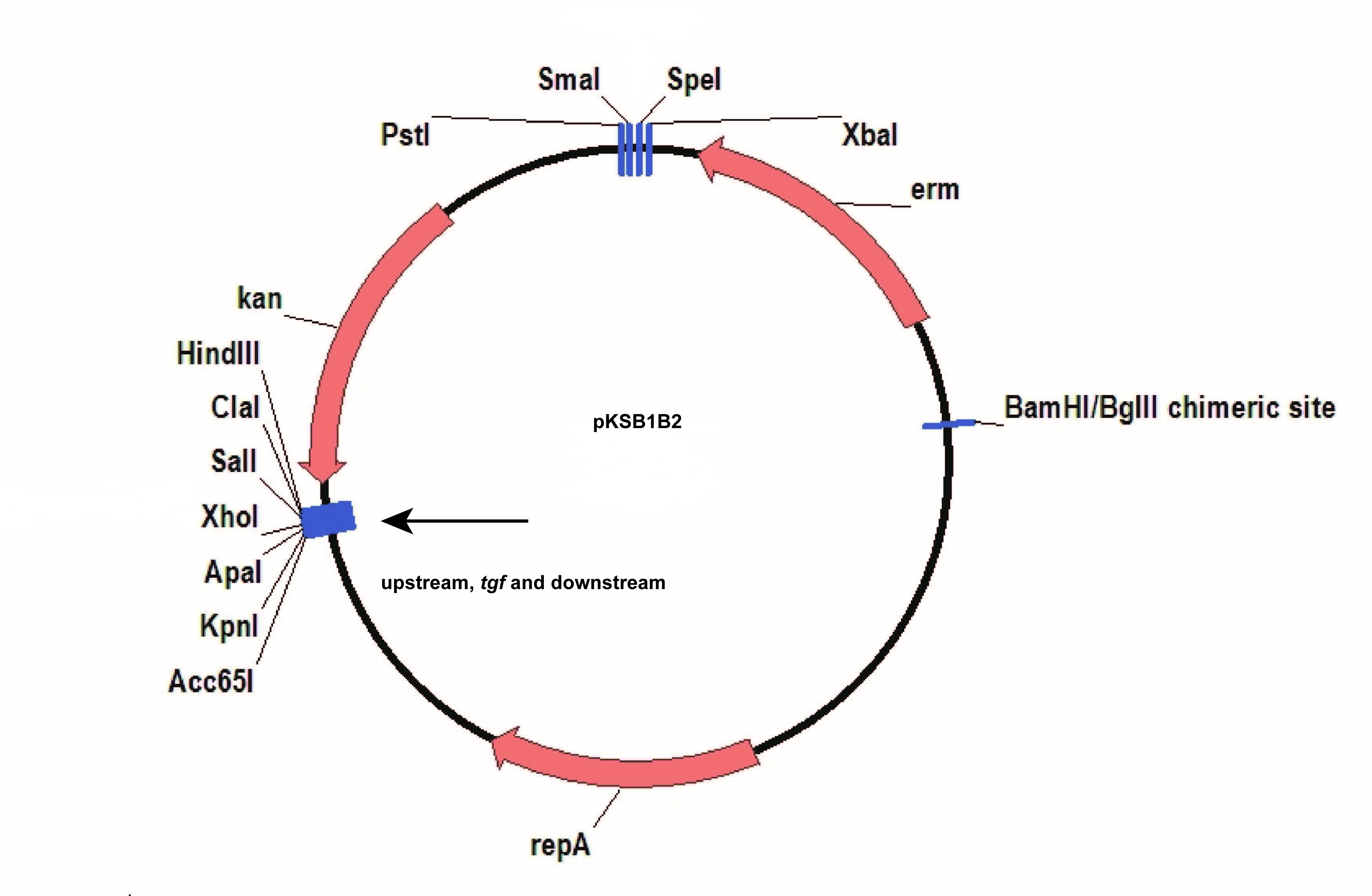


**Fig. S4** Map of plasmids used to construct point mutation and permutation mutants.


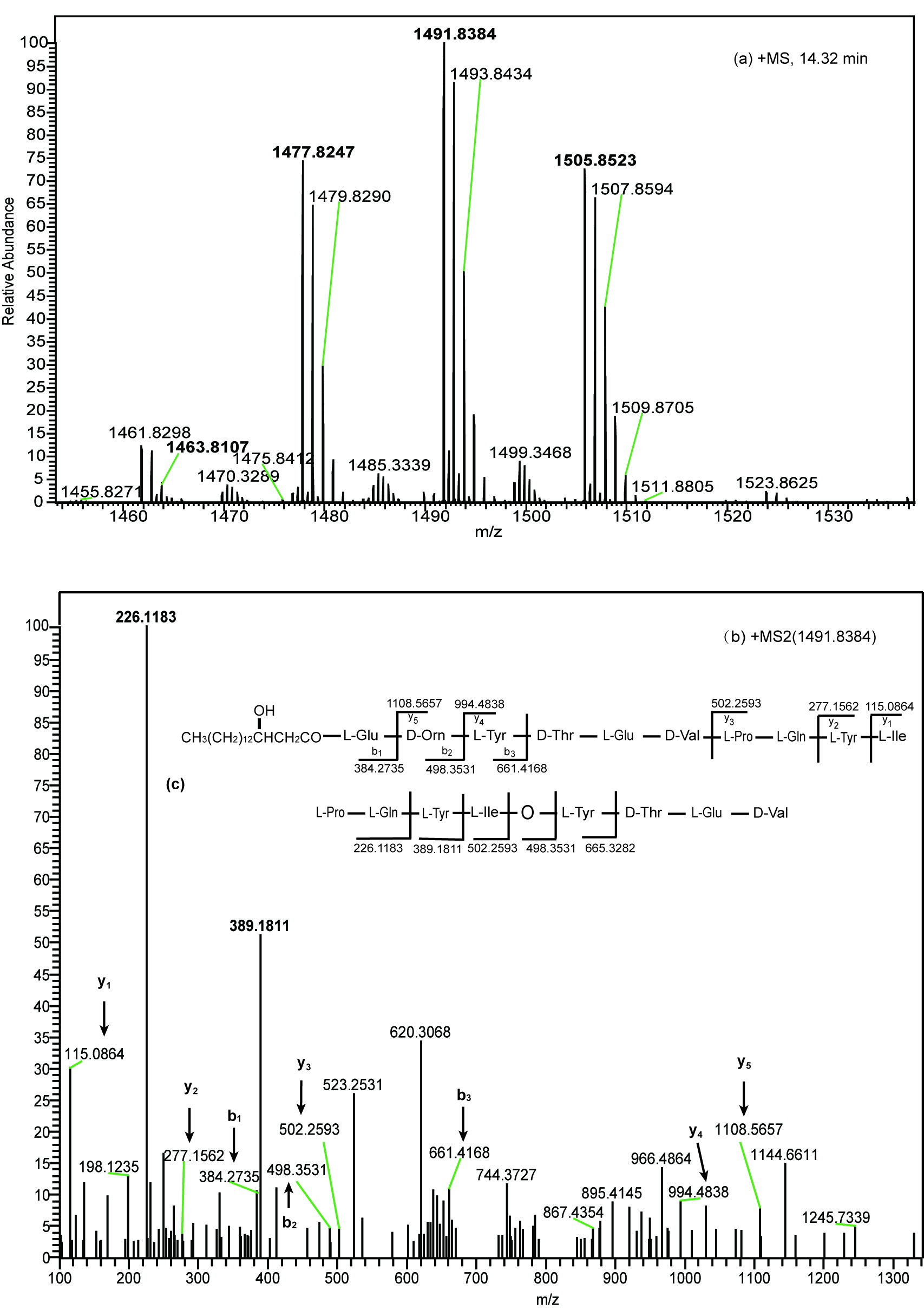


**Fig. S5** The high-resolution ESI–MS of cyclic plipastatin ions eluted at a retention time of 14.32 min (a). HCD–MS/MS of the precursor ion [M+H]^+^ at 1491.8384 m/z (b).Sequence of plipastatin with a C_12_ β-OH fatty acid chain (c).


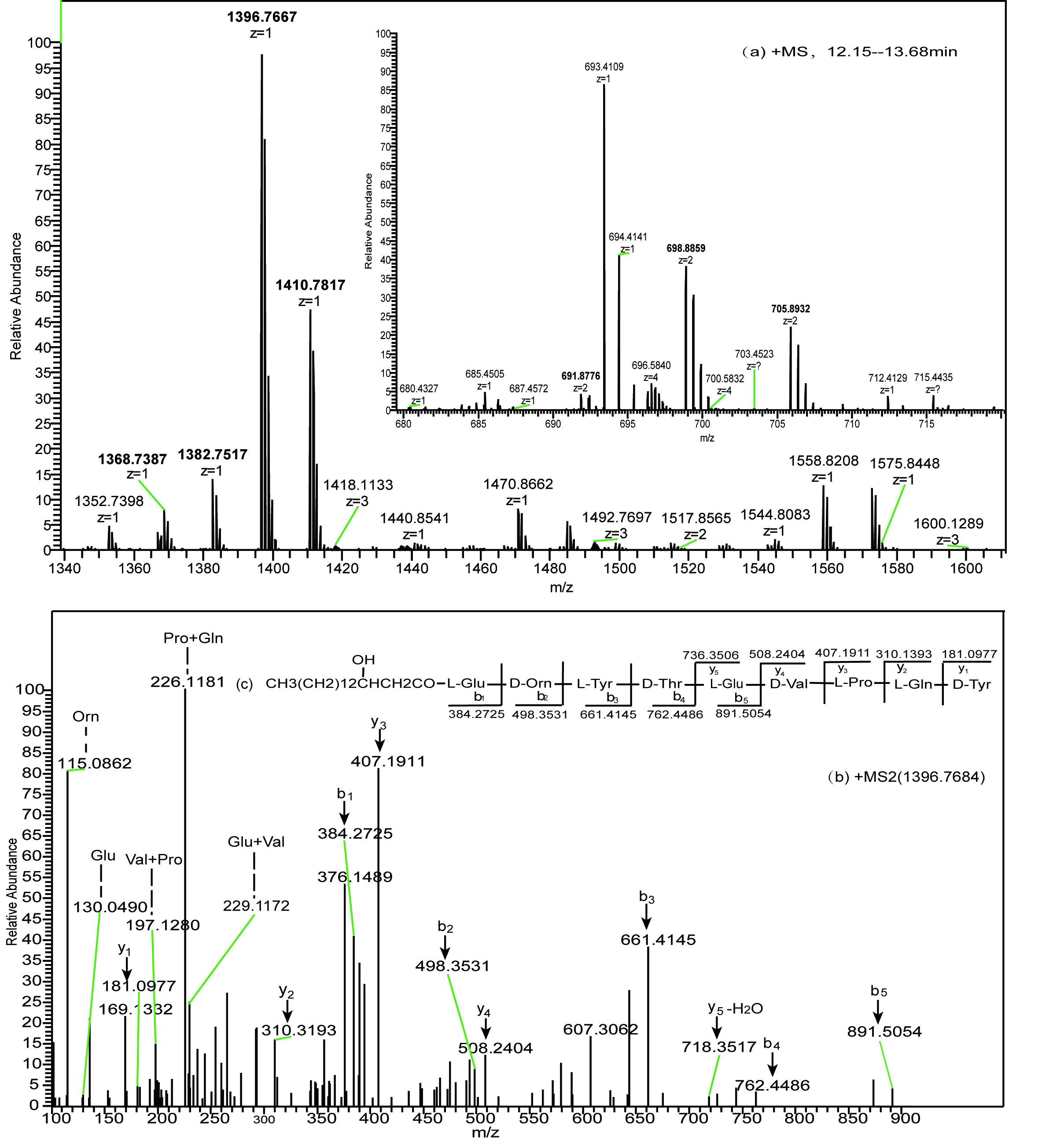


**Fig. S6.** (a) The high-resolution ESI–MS of linear nonapeptide ions eluted with RT in the range 12.15-13.68 min and (b) HCD–MS/MS of the precursor ion [M + H]^+^ at *m/z* 1,396.7667, which was confirmed as a novel nonapeptide consisting of Glu-Orn-Tyr-Thr-Glu-Val-Pro-Gln-Tyr and a C_12_ β-OH fatty acid chain (c).


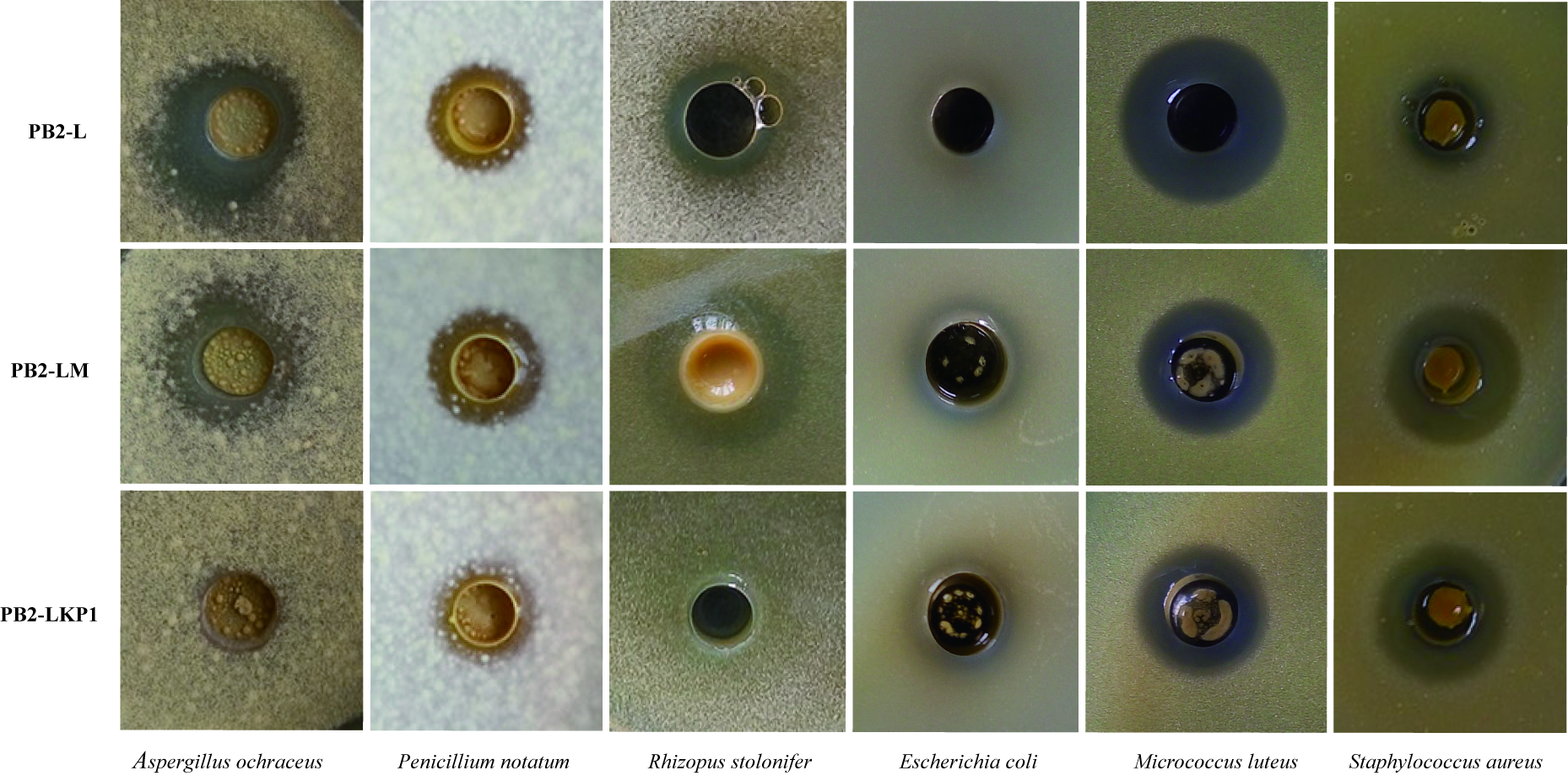


**Fig. S7** Inhibition of *Aspergillus ochraceus* CICC 2050, *Penicillium notatum* AS3.4356, *Rhizopus stolonifer* AS3.2336, *Escherichia coli* AS1.487, *Micrococcus luteus* AS1.191 and *Staphylococcus aureus* GIM 1.178, by the methanol extracts of *B. subtilis* PB2-L, *B. subtilis* PB2-LM and *B. subtilis* PB2-LKP1 fermentation broth, and the corresponding main product were the cyclic plipastatin, cyclic pentapeptide and cyclic octapeptide.
